# Gene expression differs in susceptible and resistant amphibians exposed to *Batrachochytrium dendrobatidis*

**DOI:** 10.1098/rsos.170910

**Published:** 2018-02-28

**Authors:** Evan A. Eskew, Barbara C. Shock, Elise E. B. LaDouceur, Kevin Keel, Michael R. Miller, Janet E. Foley, Brian D. Todd

**Affiliations:** 1 Graduate Group in Ecology, University of California, Davis, One Shields Avenue, Davis, CA 95616, USA; 2 EcoHealth Alliance, 460 West 34th Street – 17th Floor, New York, NY 10001, USA; 3 Department of Biology, Lincoln Memorial University, 6965 Cumberland Gap Parkway, Harrogate, TN 37752, USA; 4 Department of Pathology, Microbiology, and Immunology, School of Veterinary Medicine, University of California, Davis, One Shields Avenue, Davis, CA 95616, USA; 5 Northwest ZooPath, 654 West Main Street, Monroe, WA 98272, USA; 6 Department of Animal Science, University of California, Davis, One Shields Avenue, Davis, CA 95616, USA; 7 Department of Medicine and Epidemiology, School of Veterinary Medicine, University of California, Davis, One Shields Avenue, Davis, CA 95616, USA; 8 Department of Wildlife, Fish, and Conservation Biology, University of California, Davis, One Shields Avenue, Davis, CA 95616, USA

**Keywords:** emerging infectious disease, chytridiomycosis, comparative transcriptomics, host defences, amphibian immunity, host–pathogen interactions

## Abstract

Chytridiomycosis, the disease caused by the fungal pathogen *Batrachochytrium dendrobatidis* (*Bd*), has devastated global amphibian biodiversity. Nevertheless, some hosts avoid disease after *Bd* exposure even as others experience near-complete extirpation. It remains unclear whether the amphibian adaptive immune system plays a role in *Bd* defence. Here, we describe gene expression in two host species—one susceptible to chytridiomycosis and one resistant—following exposure to two *Bd* isolates that differ in virulence. Susceptible wood frogs (*Rana sylvatica*) had high infection loads and mortality when exposed to the more virulent *Bd* isolate but lower infection loads and no fatal disease when exposed to the less virulent isolate. Resistant American bullfrogs (*R. catesbeiana*) had high survival across treatments and rapidly cleared *Bd* infection or avoided infection entirely. We found widespread upregulation of adaptive immune genes and downregulation of important metabolic and cellular maintenance components in wood frogs after *Bd* exposure, whereas American bullfrogs showed little gene expression change and no evidence of an adaptive immune response. Wood frog responses suggest that adaptive immune defences may be ineffective against virulent *Bd* isolates that can cause rapid physiological dysfunction. By contrast, American bullfrogs exhibited robust resistance to *Bd* that is likely attributable, at least in part, to their continued upkeep of metabolic and skin integrity pathways as well as greater antimicrobial peptide expression compared to wood frogs, regardless of exposure. Greater understanding of these defences will ultimately help conservationists manage chytridiomycosis.

## 1. Introduction

Understanding and managing emerging infectious diseases is increasingly a conservation necessity [1–3]. Amphibians epitomize this problem, having recently experienced dramatic, widespread population declines [4,5]. Although amphibian declines are driven by multiple, interacting stressors [6,7], substantial amphibian biodiversity loss has been attributed to the emerging disease chytridiomycosis, caused by the aquatic fungal pathogen *Batrachochytrium dendrobatidis* (*Bd*) [8–10]. *Bd* infection can induce severe physiological disruption in amphibian hosts, leading to death [11], and the pathogen has an extremely broad host range, infecting hundreds of species across diverse amphibian families [12]. Consequently, *Bd* epidemics have driven global amphibian declines, extirpations and extinctions [13].

Given the devastating impact *Bd* has had on global amphibian biodiversity, understanding how certain species cope with *Bd* exposure and infection represents a critical knowledge gap. Some wild amphibian populations persist despite *Bd* infection [14,15], and mortality rates differ among species exposed experimentally to *Bd* [16,17]. Multiple, complementary mechanisms may explain how specific amphibian host species defend against *Bd* infection and disease pathology. For example, morphological processes like skin sloughing could help hosts resist pathogen colonization [18–20], and the diverse—yet species-specific—suite of antimicrobial peptides (AMPs) in amphibian skin mucus constitutes an innate defence against *Bd* [16,21,22]. In addition, certain amphibian skin-associated microbes produce anti-*Bd* compounds, and thus skin microbiome composition can affect *Bd* infection intensities and disease severity in exposed individuals [23,24].

The adaptive immune system is a critical vertebrate defence mechanism, yet there is conflicting evidence about whether and how adaptive immunity contributes to amphibian defences against *Bd* [25]. Some experimental studies indicate that prior exposure to killed or live *Bd* does not improve survival of frogs undergoing subsequent exposures, suggesting absent or ineffective adaptive immune memory [26,27]. There is also a notable lack of immune gene upregulation in some amphibian species exposed to *Bd* [28,29]. Other studies directly contradict these results, finding improved resistance to *Bd* as a result of prior exposure [30,31]. Variation in certain adaptive immune system components, like MHC, has also been linked to chytridiomycosis resistance [25,32–34]. However, in a further complexity, *Bd* itself may inhibit the amphibian adaptive immune system by limiting proliferation and inducing apoptosis of lymphocytes [35–37]. *Bd*'s inhibitory activity could, therefore, render an adaptive immune response ineffective and costly for amphibian hosts.

To help clarify the adaptive immune system's role in generating beneficial responses to *Bd* challenge, here we report on experimental *Bd* exposures with two amphibian host species, one susceptible to chytridiomycosis and one resistant. Specifically, we use gene expression profiling of host skin tissue, the primary site of infection, to reveal differences in immune system activation. Our model susceptible species, the wood frog (*Rana sylvatica*), is known to die from chytridiomycosis in laboratory [17] and field settings [38]. In contrast, our model resistant species, the American bullfrog (*R. catesbeiana*), is generally unaffected by *Bd* exposure [39–41] and has been implicated as a driver of global *Bd* transmission due to its invasiveness [42,43]. In addition, because different *Bd* isolates can cause different disease outcomes [17,44], we exposed our amphibian host species to two *Bd* isolates that differ in virulence [45]. Therefore, we simultaneously profiled gene expression in the two amphibian hosts as well as the two *Bd* isolates, an approach that can highlight complex host–pathogen interactions [46,47].

## 2. Material and methods

### 2.1. Study animal collection, rearing and husbandry

To obtain study animals, we first collected recently laid wood frog egg masses just after oviposition in March 2014 in Montgomery County, VA. Eggs were shipped to Davis, CA where we housed them in 10-gallon buckets filled with deionized (DI) water reconstituted using powdered water conditioner (R/O Right, Kent Marine, Franklin, WI). Bubblers maintained dissolved oxygen (DO). Upon hatching, we transferred wood frog tadpoles to previously established mesocosms at densities of 50 tadpoles per mesocosm. We used a total of six mesocosms for this study; each was a 300-gallon cattle tank that we seeded with 1 kg of leaf litter and 4 l of local pond water before filling the remainder of the tank with DI water. All mesocosms were established approximately four weeks prior to the introduction of tadpoles, and they were maintained inside a greenhouse in order to moderate temperature. Mesocosm water temperature ranged from approximately 22 to 26**°**C, and we outfitted all mesocosms with air stones to supplement DO. To prevent study animals from escaping and to prevent colonization by unwanted fauna, we placed mesh netting over each mesocosm. We constructed small Styrofoam floats and placed one in each mesocosm to provide metamorphosing animals with perches to avoid drowning. We monitored mesocosms daily for water level, temperature, DO and the presence of any metamorphosed animals, which were removed from the mesocosms and transferred to an animal care room. When animals began to metamorphose, we also hung partially submerged minnow traps from the sides of the mesocosms to aid in their capture.

In contrast to wood frogs, which can metamorphose within 6−8 weeks after hatching, American bullfrogs often overwinter as tadpoles [48]. Thus, we collected late-stage American bullfrog tadpoles to efficiently raise them to metamorphosis for use in our study. During summer 2014, we collected wild American bullfrog tadpoles from two locations in their introduced range in California: Stone Lakes National Wildlife Refuge in Elk Grove, CA and Putah Creek Riparian Reserve in Davis, CA. We visually inspected mouthparts of all captured American bullfrog tadpoles and only kept animals with normal mouthpart pigmentation and jaw sheath structure to minimize the likelihood of introducing *Bd*-infected animals into our mesocosms [49]. Because wood frog tadpoles completed metamorphosis quickly, we transferred wild-caught American bullfrog tadpoles to the same (now unoccupied) mesocosms used to rear wood frogs. We added an additional 1 kg of leaf litter to each mesocosm prior to introducing American bullfrogs. Subsequent methods for rearing American bullfrogs from tadpoles were identical to those used for wood frogs. Wood frog and American bullfrog tadpoles were never housed together.

Upon metamorphosis, we housed all animals individually in 5.7 l lidded plastic containers. At the time of removal from mesocosms and transfer to individual housing, wood frogs averaged approximately 20 mm snout–urostyle length (SUL) and weighed approximately 0.79 g, while American bullfrogs were approximately 40 mm in SUL and weighed approximately 7.82 g on average. Each animal's container was tilted to provide aquatic habitat (reconstituted DI water) at one end and terrestrial habitat at the other. A plastic cup modified into a shelter structure provided habitat enrichment. The animal care room was maintained at 18**°**C on a 12 L : 12 D cycle. We conducted daily health checks to ensure animals were alert and maintained a righting reflex. Clean nitrile gloves were used to handle each individual when providing animal care. We fed animals twice weekly, with each meal consisting of 2−3 appropriately sized crickets dusted with multivitamins (ReptiVite with D3, Zoo Med Laboratories, San Luis Obispo, CA) and calcium supplement (Rep-Cal Research Labs, Los Gatos, CA). We thoroughly rinsed tanks and refilled them with clean water twice weekly on the day after each feeding. After *Bd* exposures were applied, we attended to control animals before exposed animals during all animal care procedures to reduce the possibility of contamination among treatment groups.

### 2.2. Experimental *Bd* exposures and animal monitoring procedures

Three days before experimental *Bd* exposures, and weekly thereafter, we weighed (g) and measured (SUL in mm) all study animals. In addition, we assayed all animals by quantitative polymerase chain reaction (qPCR) (see detailed methods below) for *Bd* prior to exposure treatments. Animals used in this study were assumed to be *Bd*-naive because they were collected as eggs or tadpoles, raised to metamorphosis in captivity, and all individuals of both study species tested *Bd*-negative prior to experimental exposures. To collect samples for *Bd* testing, we swabbed animals with a sterile rayon swab (MW113, Medical Wire and Equipment, Wiltshire, UK) for a total of 25 strokes: five each on the ventral surface, the ventral side of each thigh, and on each foot [45]. Swab samples were stored at −20**°**C until further analysis.

For exposures, we used two different isolates of *Bd* known to differ in virulence: low-virulence Carter Meadow *Bd* (CM *Bd*) and high-virulence Section Line *Bd* (SL *Bd*). See Piovia-Scott *et al.* [45] and Eskew *et al.* [41] for more information on these *Bd* isolates. For this experiment, both *Bd* isolates were revived from low-passage stocks frozen in liquid nitrogen approximately three months prior to *Bd* exposures. To prepare exposure inoculum, we cultured *Bd* zoospores in TGhL broth and, following filtration, quantified zoospore density using a haemocytometer as in Piovia-Scott *et al.* [45].

We intended to include a total of 210 animals in the study, incorporating 35 CM *Bd*-exposed, 35 SL *Bd*-exposed, and 35 sham control individuals for each of the two study species. However, we were only able to expose five American bullfrogs to CM *Bd* due to insufficient quantities of that isolate. Our study also included a set of eight wood frogs that had been previously exposed to SL *Bd* and survived (hereafter the ‘PE SL *Bd*’ treatment). Specifically, these animals had undergone between one and four SL *Bd* exposures in the weeks prior to the experiment with exposure dosage varying from approximately 10^5^ to approximately 3 × 10^6^ total zoospores. In the results reported here, PE SL *Bd* study animals were then re-exposed using the same protocols as for naive individuals exposed to SL *Bd*. Thus, our experiment consisted of 188 total animals (electronic supplementary material, table S1). We randomly assigned study animals to experimental treatments and made minor adjustments to ensure there were no initial differences in mass or size among treatment groups within species. At the time of our full *Bd* exposure treatments, wood frogs were approximately 5.5 months post-metamorphosis, and American bullfrogs were approximately 1.5−4.5 months post-metamorphosis.

Because of the differences in body size between the two frog species, we exposed American bullfrogs to three times the amount of *Bd* used for wood frog exposures. This scaling factor was chosen to provide for approximately equal exposure per surface area, calculated by taking the mean estimated surface area of each study species. We exposed each wood frog to approximately 7.5 × 10^6^ total zoospores and each American bullfrog to approximately 2.25 × 10^7^ total zoospores. These doses are similar to those used in previous studies involving the same amphibian host species and *Bd* isolates, which ranged from approximately 10^5^ to approximately 10^8^ total zoospores [18,39,45]. We pipetted the exposure broth (TGhL broth + quantified *Bd* zoospores) directly onto the venter to ensure skin contact. Sham control animals were exposed to TGhL broth without *Bd*. Exposures lasted overnight; we housed wood frogs in 100 × 15 mm Petri dishes with a total volume of 20 ml liquid (exposure broth + reconstituted DI water) and American bullfrogs in plastic Ziploc containers (237 ml volume) with 50 ml liquid. Containers and exposure volumes were chosen to restrict frog movement and ensure constant contact with *Bd*. At the end of the overnight exposure period, all animals were returned to their usual enclosures along with the contents of their exposure containers, allowing for further opportunity for *Bd* contact until housings were cleaned four days post-exposure.

We monitored the health of all study animals daily for 49 days after exposure. As previously indicated, we weighed and measured all animals on a weekly basis. At the same weekly interval, we also swabbed all experimentally exposed animals for *Bd*, along with a random selection of 5−10 control animals of each study species. We humanely euthanized animals via overdose of MS-222 if they failed to exhibit a righting reflex. *Bd* swab samples were collected from these study animals prior to euthanasia. Carcasses of select animals were kept cool for approximately 3−12 h prior to formalin-fixation for histological analysis.

### 2.3. Tissue harvesting

To collect tissues for gene expression analyses, we randomly sacrificed five animals of each species from each treatment at three time points: 3, 7 and 10 days post-exposure (electronic supplementary material, table S2). Because only five American bullfrogs were exposed to CM *Bd*, we could only collect samples from the first time point for that treatment. For PE SL *Bd*-exposed wood frogs, we collected samples from four animals at 3 days post-exposure and from three animals at 7 days post-exposure. Prior to euthanizing, we weighed, measured and swabbed all animals. For the animals from which we collected tissue samples, we euthanized using a combination of decapitation and pithing because we were concerned that cutaneous exposure to MS-222 might influence gene expression in the skin. Immediately after euthanasia, we used sterile instruments to harvest approximately 20–30 mg of ventral skin tissue from each study animal. We promptly homogenized tissue samples in 1.5 ml TRIzol reagent using a hand-held Omni TH tissue homogenizer (Omni International, Kennesaw, GA). The homogenizer probe was rinsed thoroughly in 70% ethanol and multiple washes of molecular grade water between tissue samples. We stored all tissue samples at −80**°**C.

### 2.4. Survival and body mass analyses

To test for effects of *Bd* exposure on frog survival, we used the R package ‘survival’ [50]. We generated species-specific survival datasets, fitted survival curves corresponding to each treatment group within a species, and used the function ‘survdiff’ to test for differences among those survival curves. Our analysis used censoring to account for removal of animals that were sacrificed for tissue harvesting.

Even when chytridiomycosis does not cause death, it may still have significant negative effects on host physiology and body condition [44,51–54]. Therefore, we analysed frog body mass data collected throughout the experiment to evaluate sublethal effects of pathogen exposure. We fitted a linear mixed-effects model for each treatment group within each species. In these models, frog body mass was the continuous response variable, days post-exposure (i.e. time course of the experiment) was a categorical predictor, and study animal identity was included as a varying effect. Thus, these mixed models account for the inherent structure of our data, wherein repeated mass measurements were collected from the same individuals over time. We fitted models using the ‘lmer’ function within the R package ‘lme4’ [55] and calculated 95% confidence intervals (CIs) on model parameters using the ‘precis’ function from the ‘rethinking’ package [56]. We coded each model such that the intercept parameter represented pre-exposure body mass. Hence, other parameters reflected the difference between body mass measured at a later experimental time point and pre-exposure body mass. We interpreted cases where the 95% CI for one of these parameters did not overlap zero as evidence for a significant change in frog body mass relative to the pre-exposure time point.

### 2.5. Infection prevalence and load via quantitative polymerase chain reaction

We used a well-established qPCR assay as our primary means of determining *Bd* infection status [57,58]. Specifically, we used the qPCR protocols of Piovia-Scott *et al.* [45] to quantify *Bd* loads on amphibian skin swabs; for this assay, samples are run in singlicate [59] and raw qPCR quantifications are multiplied by 160 to account for dilutions occurring during DNA extraction, thus producing an estimate of *Bd* zoospore equivalents (ZE) from each sample. This qPCR assay allowed us to estimate *Bd* infection loads on individual animals and determine infection prevalence within experimental treatment groups.

### 2.6. Infection quantification via histology

Some authors suggest that qPCR could be, paradoxically, a misleading indicator of biologically relevant chytridiomycosis disease status because it is capable of detecting extremely small amounts of DNA [60]. In addition, qPCR data from skin swab samples may not accurately quantify an individual's true infection burden [61]. Thus, to help mitigate these concerns, we supplemented our qPCR infection load data with histological examination of skin tissues.

We performed histology on 90 samples taken from a subset of study animals. These histology samples represent individuals that were sacrificed for tissues (*n* = 67) and those that were euthanized when they began to show morbidity during experimental monitoring (*n* = 23). Legs and feet from sampled individuals were trimmed for histology. For each case, four longitudinal sections from the pelvic limbs were placed in a single cassette; the feet were placed whole in an additional cassette. Trimmed tissues were processed for routine histology, sectioned at 5 µm, and stained with Gomori methenamine silver.

Slides of legs and feet were evaluated by a board-certified veterinary pathologist (E.E.B.L.). In each case, the entire skin surface from both legs and feet was evaluated at 200× magnification for the presence of thalli. The number of 200× fields examined for each case ranged from 48 to 122, depending on the size of the animal (e.g. American bullfrogs tended to have a larger skin surface area than wood frogs), size of the trimmed sample, and amount of artefact (e.g. there was rare separation or loss of skin during processing). Each 200× field was scored as having a low, medium or high burden of infection, or as negative for infection. Low burden scores represented 200× fields with 1−19 thalli, medium scores designated 20−60 thalli per field, and high scores were characterized by greater than 60 thalli per field. Each case was then assigned an infection score based on the number of negative, low, medium, or high fields (electronic supplementary material, table S3). Infection scores were calculated as follows. First, the percentages of negative, low, medium, and high burden fields were determined for each case. Next, the percentages were scaled for the severity of infection by multiplying percentages of negative burden fields by zero, low by one, medium by two, and high by three. Then, the scaled percentages were summed and rounded to the nearest whole number to result in a final infection score. For example, if one case had 10% negative fields, 65% low fields and 25% medium fields, the infection score would be 10(0) + 65(1) + 25(2) = 115. Based on this system, possible infection scores ranged from 0 (100% negative fields) to 300 (100% high fields).

### 2.7. RNA isolation, library preparation and sequencing

We isolated RNA from ventral skin tissues homogenized in TRIzol using Zymo Research Direct-zol kits with an on-column DNase I digestion step. We followed the manufacturer's standard extraction protocol, except that we extended DNase I digestion time to 25 min and decreased the final elution volume to 30 µl. Following RNA extraction, we generated sequencing libraries from mRNA transcripts from each tissue sample using NEBNext Ultra RNA kits (New England BioLabs, Ipswich, MA) and a poly(A) selection strategy. Libraries were individually barcoded and pooled for high-throughput sequencing. All sequencing was conducted on the Illumina HiSeq 3000 platform at the UC Davis Genome Center (Davis, CA) using the 100 bp paired-end mode. We obtained reads from 87 RNA-seq libraries using a total of 11 sequencing lanes.

### 2.8. RNA-seq read processing, transcript quantification and gene expression analyses

After generating RNA-seq reads from ventral skin tissue samples, we used FastQC software (Babraham Bioinformatics, Cambridge, UK) to visualize and verify read quality. We used the experimental High Throughput Sequencing (expHTS) pipeline to perform consistent read processing for all of our RNA-seq samples [62]. The expHTS pipeline includes contamination screening, PCR duplicate removal, and quality trimming steps.

In lieu of aligning to different reference transcriptomes for each host species, which would complicate comparisons between species, we aligned all RNA-seq reads to a common host transcriptome. Specifically, we used a previously published transcriptome for *R. clamitans* [63], a congener to our two study species. Robertson & Cornman [63] also reported 11 manually curated, candidate AMP sequences from *R. clamitans*. Because AMPs can constitute an important amphibian defence against *Bd* [16,21], we included these sequences in our *R. clamitans* reference transcriptome.

A number of sequencing studies have recovered pathogen sequences as either a primary aim [46,47] or secondary aspect [64] of their investigations of host organisms. While some previous gene expression studies examining amphibian responses to *Bd* infection have sought to exclude potential *Bd*-derived sequences from analysis [65], others have explicitly called for such a ‘dual RNA-seq’ approach to study host–pathogen interactions in the amphibian–*Bd* system [66]. Following these recommendations, we sought to characterize any *Bd*-derived sequence that might be present in sampled host tissues using the reference *Bd* transcriptome generated by the Broad Institute (Cambridge, MA). We, therefore, concatenated the *R*. *clamitans* and *Bd* reference transcriptomes, allowing for the full set of host- and pathogen-associated contigs to be present during quantification. We quantified expHTS-processed RNA-seq reads from each sample against this concatenated host–pathogen reference transcriptome using Salmon [67]. Read abundance estimates from Salmon were rounded to the nearest whole number and treated as counts in downstream analyses.

We used the R software package ‘edgeR’ to conduct differential expression analyses [68–70]. First, we created host- and pathogen-specific datasets to be analysed in parallel because we did not want to include *Bd* contigs in analyses of host gene expression and vice versa. Using the host-specific dataset, we then filtered out contigs with low expression; we only kept contigs having read counts greater than five in at least seven RNA-seq samples. We then combined experimental main effects (host species, exposure treatment and time point) into one factor and constructed a design matrix where each unique combination of main effects (e.g. ‘wood frog–SL *Bd*–day 3’) was represented by a single coefficient. We fitted our data using this design matrix and the ‘edgeR’ function ‘glmQLFit’, which implements a quasi-likelihood negative binomial generalized linear model. Following model fitting, we tested for differences in host gene expression by specifying pairwise contrasts between *Bd* treatment groups and time-matched controls within species (e.g. ‘wood frog–SL *Bd*–day 3’ versus ‘wood frog–control–day 3’). We made differential expression calls using the quasi-likelihood *F*-test method [71], and we considered contigs with false discovery rate-corrected *p*-values ≤0.05 to be differentially expressed.

We also wanted to directly compare species' responses to *Bd* exposure. Therefore, we first plotted wood frog responses to *Bd* exposure (i.e. log2 fold change of each contig relative to controls) at all relevant time points against the equivalent American bullfrog responses. We quantified similarity in species' responses to *Bd* exposure using the Pearson correlation coefficient. To more formally test for differences in species' responses to *Bd*, we turned to the previously described gene expression model. In particular, we specified contrasts that compared wood frog responses to a given *Bd* isolate at a given time point (e.g. ‘wood frog–SL *Bd–*day 3’ versus ‘wood frog–control–day 3’) to American bullfrog responses to the same isolate at the same time point (e.g. ‘American bullfrog–SL *Bd–*day 3’ versus ‘American bullfrog–control–day 3’). We then conducted differential expression calling as before. These contrasts explicitly compare species-level responses to *Bd* exposure and are equivalent to results that could be obtained from a model specified with full interactions among main effects (i.e. host species × exposure treatment × time point).

To test for differences in *Bd* gene expression, we used the pathogen-specific RNA-seq dataset and filtered out low expression contigs as previously described. We then filtered the dataset further to only include *Bd* contigs that had read counts less than 10 across all control samples. The use of *Bd* contigs that were extremely rare or absent in control samples increases the likelihood that these sequence reads derived from *Bd* transcripts. For this subset of contigs, we constructed a design matrix and fitted a generalized linear model as before. We then specified pairwise SL *Bd* versus CM *Bd* and PE SL *Bd* versus SL *Bd* contrasts within host species and time points. Differential expression calling was conducted as previously described.

We visualized host-associated gene expression data using multiple methods. First, we constructed multidimensional scaling plots of the read count data for all 87 RNA-seq samples using the R package ‘limma’ [72]. Next, we used Venn diagrams to visualize overlap in differentially expressed contig sets, making diagrams to compare all treatment–control pairwise contrasts within each host species and time point. To more directly compare the two host species' responses to *Bd*, we used heatmaps. Where applicable, we generated heatmaps representing three different contig sets for both CM *Bd* and SL *Bd* samples: contigs that were differentially regulated in either host species at any time point (relative to controls), contigs that showed a common response to *Bd* exposure (i.e. differential regulation in the same direction) in both host species at any time point, and contigs that showed a different response to *Bd* exposure between host species at any time point. Heatmaps were produced using the ‘heatmap.2’ function of the ‘gplots’ package, which defaults to Euclidean distances and complete linkage for clustering [73]. Finally, we plotted read counts for specific contigs of interest, controlling for differences in sequencing depth among samples by scaling raw counts to counts per million.

We used the Trinotate pipeline to generate functional annotations for our contigs [74]. Trinotate integrates, among others, BLAST [75] and Gene Ontology (GO) [76] tools. We used the resulting functional information to perform GO term enrichment analyses on selected contig sets using the ‘GOstats’ package in R [77].

## 3. Results

### 3.1. Survival and body mass of study animals

**Figure 1.**
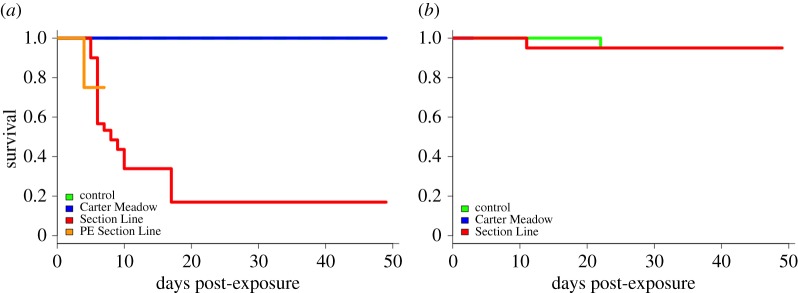
Survival curves for wood frogs (*a*) and American bullfrogs (*b*) following experimental *Bd* exposure. Green lines represent frogs treated with a sham control (*n* = 35 wood frogs, 35 American bullfrogs), blue lines are those in the CM *Bd* treatment group (*n* = 35 wood frogs, 5 American bullfrogs), red lines represent frogs in the SL *Bd* treatment group (*n* = 35 wood frogs, 35 American bullfrogs), and the orange line shows wood frogs that were previously exposed to SL *Bd* then re-exposed (*n* = 8). In (*a*), the line representing control animals is obscured as this treatment group had 100% survival throughout the study. In (*b*), the line representing CM *Bd*-exposed animals is obscured as these five animals survived for three days following *Bd* exposure but were then sacrificed for tissue harvesting.

*Bd* exposure effects on wood frog survival were strongly dependent on isolate. SL *Bd*-exposed wood frogs were severely affected, such that only one of 35 animals initially in this treatment survived until the end of the study (figure 1*a*). Note however that here, as in all treatments, animals were removed from the experiment not only due to morbidity but also because of destructive sampling for tissue harvesting, which was accounted for using censoring in the survival analyses. As a result, the set of animals for which morbidity could be observed throughout the experiment did not remain constant at the initial treatment sample size. Wood frogs in the PE SL *Bd* treatment (*n* = 8) also had reduced survival. Following the removal of four of these animals for tissue harvesting at 3 days post-exposure, one of the remaining four animals experienced morbidity prior to tissue harvesting at 7 days post-exposure, when the final three individuals were removed. In contrast, no CM *Bd*-exposed wood frogs (*n* = 35) died. As expected, control wood frogs (*n* = 35) also had high survival (100%). Owing to the dramatic effect of SL *Bd* exposure relative to the other treatments, there were significant differences in wood frog survival among treatment groups (*χ*^2 ^= 56.9, df = 3, *p* < 0.001).

In contrast, American bullfrog survival did not differ among experimental treatments (*χ*^2 ^= 0, df = 1, *p* = 0.986; figure 1*b*). Despite being exposed to greater absolute numbers of *Bd* zoospores than wood frogs, American bullfrogs had high survival across all treatments, with only one case of apparently incidental mortality occurring in each of the control (*n* = 35) and SL *Bd*-exposed (*n* = 35) groups. All CM *Bd*-exposed American bullfrogs (*n* = 5) survived until the first tissue harvesting time point at 3 days post-exposure, when all individuals were removed from that treatment group.

**Figure 2.**
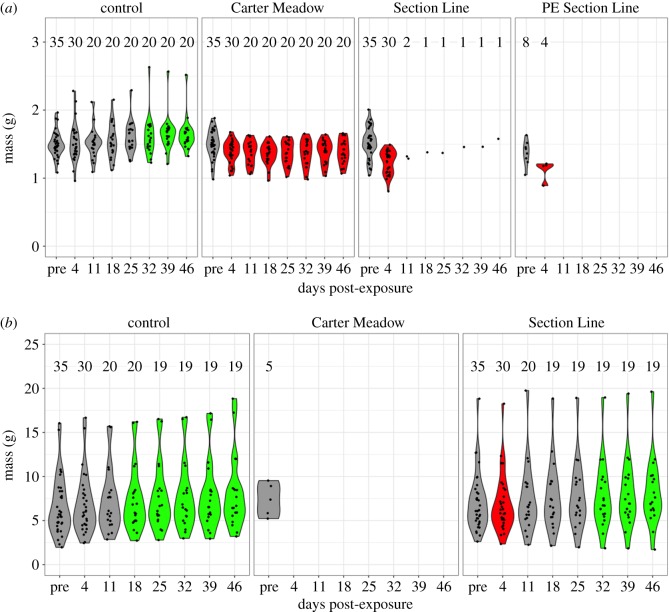
Violin plots showing body mass of wood frogs (*a*) and American bullfrogs (*b*) following experimental *Bd* exposure. Body mass data were collected from all surviving study animals on a weekly basis, starting three days prior to experimental exposure treatments (the ‘pre’ time point). Jittered black points in the violin plots represent the individual body mass measurements, and the numbers above each plot explicitly list the sample size at each time point. Violin plots with green fill represent time points where body mass is significantly greater than the pre-exposure body mass for that treatment, whereas red fill indicates a significant decrease in body mass. We fitted body mass data using linear mixed-effects models, and significance was inferred when the 95% confidence interval for a parameter estimate did not overlap zero. Violin plots with grey fill represent time points that are statistically identical to the pre-exposure time point.

Body mass of study animals changed over time, with contrasting patterns across treatments (figure 2). Body mass of control wood frogs increased slightly over time, becoming significantly greater than pre-exposure values at 32 days post-exposure and for all time points thereafter (absolute change in mean body mass from pre-exposure to day 46: +0.15 g; relative change: +10.1%). In contrast, both CM and SL *Bd*-exposed wood frogs showed significant decreases in body mass immediately after exposure. Patterns of body mass change for American bullfrogs were qualitatively similar. For example, control American bullfrogs showed significant increases in body mass late in the experiment (absolute change in mean body mass from pre-exposure to day 46: +1.57 g; relative change: +23.88%). American bullfrogs exposed to SL *Bd* showed a significant decrease in body mass immediately following exposure, mirroring the pattern seen in *Bd*-exposed wood frogs. However, these American bullfrogs then began to gain mass such that they were significantly heavier at the final three measurement time points than they were pre-exposure.

### 3.2. Infection prevalence and load via quantitative polymerase chain reaction

**Figure 3.**
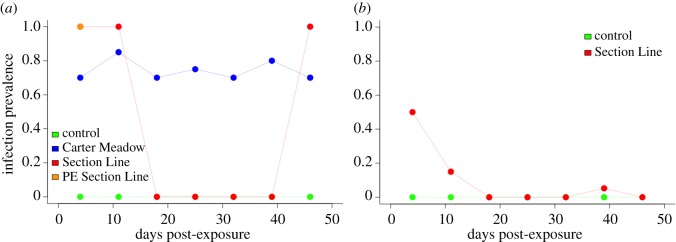
*Bd* infection prevalence in wood frogs (*a*) and American bullfrogs (*b*) following experimental *Bd* exposure. We quantified infection with a qPCR assay, using frog skin swab samples as starting material (see main text for further detail). Green lines represent frogs treated with a sham control, blue lines are those in the CM *Bd* treatment group, red lines represent frogs in the SL *Bd* treatment group, and orange shows wood frogs that were previously exposed to SL *Bd* then re-exposed. Data from harvesting dates (3, 7 and 10 days post-exposure) are not shown in order to maintain a presentation of our weekly sampling data consistent with figure 2 and because harvesting dates have low sample sizes (*n *≤ 5) relative to weekly time points. Thus, data from CM *Bd*-exposed American bullfrogs do not appear in (*b*) (all animals in this treatment group were sacrificed for tissue harvesting at 3 days post-exposure). Sample sizes for these analyses are given in the electronic supplementary material, table S1.

Following experimental *Bd* exposure, wood frogs showed evidence of infection via qPCR assays of skin swab samples, whereas American bullfrogs largely appeared to resist infection (figure 3). Exposure to SL *Bd* resulted in 100% infection prevalence in wood frogs at four and 11 days post-exposure (figure 3*a*). Prevalence dropped to zero from days 18–39 post-exposure. Only one study animal remained in the SL *Bd* treatment group at this point in the experiment, and it consistently tested *Bd*-negative during this time. After appearing to have cleared *Bd* infection, the sole surviving wood frog in the SL *Bd* treatment again tested positive for *Bd* at 46 days post-exposure (see [78] for further discussion of the potential for variation in *Bd* testing results within individuals). All PE SL *Bd* wood frogs tested positive for *Bd* four days post-exposure. Despite suffering no mortality during the experiment, CM *Bd*-exposed wood frogs were consistently infected, with prevalence ranging from 70 to 85% throughout the study. Prevalence among SL *Bd*-exposed American bullfrogs peaked at 50% four days post-exposure then rapidly declined and remained low for the duration of the experiment (figure 3*b*). Only five American bullfrogs were exposed to CM *Bd*, but all of these study animals were *Bd*-positive at the first tissue harvesting time point 3 days post-exposure. Control wood frogs and American bullfrogs remained *Bd*-negative throughout the course of the experiment.

**Figure 4.**
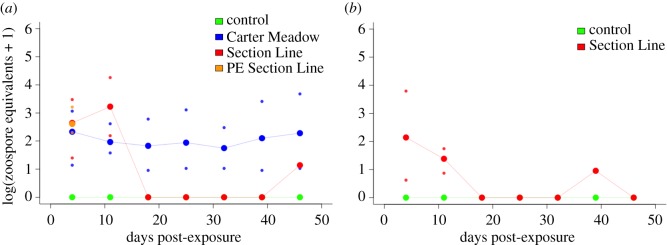
Mean *Bd* infection loads from *Bd*-positive wood frogs (*a*) and American bullfrogs (*b*) following experimental *Bd* exposure. We quantified infection with a qPCR assay, using frog skin swab samples as starting material (see main text for further detail). Individual infection loads were estimated in terms of *Bd* zoospore equivalents (ZEs). For plotting, we added one to these ZE values, performed a log10-transformation, and then averaged infection loads of *Bd*-positive animals at each time point within treatment groups. If there were no *Bd*-positive animals at a particular time point, a value of zero is displayed. Where applicable, minimum and maximum infection load values are also shown as small dots. Green lines represent frogs treated with a sham control, blue lines are those in the CM *Bd* treatment group, red lines represent frogs in the SL *Bd* treatment group, and orange shows wood frogs that were previously exposed to SL *Bd* then re-exposed. Data from harvesting dates (3, 7 and 10 days post-exposure) are not shown in order to maintain a presentation of our weekly sampling data consistent with figure 2 and because harvesting dates have low sample sizes (*n *≤ 5) relative to weekly time points. Thus, data from CM *Bd*-exposed American bullfrogs do not appear in (*b*) (all animals in this treatment group were sacrificed for tissue harvesting at 3 days post-exposure). Sample sizes for these analyses are given in the electronic supplementary material, table S1.

Average *Bd* load of *Bd*-positive animals also differed depending upon host species and *Bd* isolate (figure 4). Within wood frogs, SL *Bd* exposure resulted in heavy infections, with mean *Bd* loads of approximately 1000 ZE at both four and 11 days post-exposure. In contrast, CM *Bd* exposure caused less intense infections in wood frogs; mean *Bd* loads were approximately 100 ZE in *Bd*-positive frogs from this treatment group for the duration of the experiment. Infection loads of SL *Bd*-exposed American bullfrogs were lower than for wood frogs exposed to the same *Bd* isolate. Mean load in these animals peaked at approximately 100 ZE at four days post-exposure before declining.

### 3.3. Infection quantification via histology

**Figure 5.**
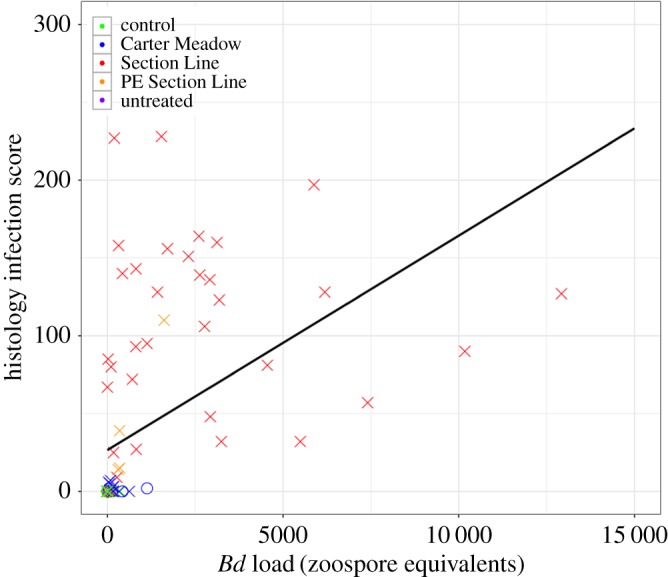
Histology infection scores versus *Bd* infection loads via qPCR for 90 frogs. Histology infection scores represent a measure of infection load, and possible scores ranged from 0–300. *Bd* infection load was also evaluated with a qPCR assay, using frog skin swab samples as starting material (see main text for further detail). Samples subject to both histology and qPCR analyses were derived from control (*n* = 12 wood frogs, 11 American bullfrogs), CM *Bd*-exposed (*n* = 13 wood frogs, 5 American bullfrogs), SL *Bd*-exposed (*n* = 32 wood frogs, 10 American bullfrogs), previously exposed SL *Bd* (*n* = 6 wood frogs), and untreated (*n* = 1 American bullfrog) study animals. For each individual, *Bd* infection load via qPCR represents data derived from the swab sample collected closest to the time of euthanasia. Data from wood frogs are shown as X's, and data from American bullfrogs are shown as O's. A best fit line is plotted in black. The Pearson correlation coefficient between the two infection quantification methods is 0.49.

Histology infection scores were positively correlated with qPCR infection loads (Pearson correlation coefficient = 0.49; figure 5 and electronic supplementary material, table S3). Observed histology scores ranged from 0 to 228, and mean scores were the highest for SL *Bd*-exposed wood frogs (*x* = 110 for this subset). Comparing data from histology and qPCR suggests qPCR is the more sensitive detection method. Of the 47 samples with a histology infection score of 0, 15 had a qPCR result greater than 0, indicating potential false negative results from histology. In contrast, among the 33 samples scoring negative for infection via qPCR, only one had a histology infection score greater than 0. These results are unsurprising given that a variety of factors, including patchy distribution of infection, are expected to reduce the sensitivity of histology as a *Bd* detection method [79].

However, discrepancies between qPCR and histology results could also be interpreted as evidence for qPCR detection of environmental *Bd* DNA (i.e. false positive results), with histology better representing the true infection status of study animals. Among *Bd*-exposed wood frog samples undergoing histological analyses, 48 were *Bd*-positive via qPCR. Of these, eight (16.7%) had histology scores of 0. In contrast, among *Bd*-exposed American bullfrog samples undergoing histological analyses, seven were *Bd*-positive via qPCR, and, of these, five (71.4%) had histology scores of 0. Although a limited sample size, these comparisons, and the totality of evidence from both qPCR and histology, suggest that whereas wood frogs were readily infected with *Bd*, many American bullfrogs resisted infection entirely or only manifested mild infections after exposure.

### 3.4. RNA-seq overview

Following read processing and quality trimming using expHTS, our RNA-seq dataset consisted of 1 078 158 711 reads across 87 ventral skin tissue samples. Read numbers from individual samples ranged from 631 487 to 38 718 641. The concatenated *R. clamitans*-*Bd* transcriptome was composed of 59 068 contigs, 22 940 (38.8%) of which were successfully annotated with GO terms using the Trinotate pipeline. Of note, 50 249 of the concatenated reference transcriptome contigs came from *R. clamitans*, and 8819 came from *Bd*. Mapping rates to the concatenated transcriptome were similar between study species: wood frog mapping rates ranged from 69.0% to 77.3%, while those of American bullfrogs ranged from 68.4% to 81.5%.

### 3.5. Host gene expression—overview

**Figure 6.**
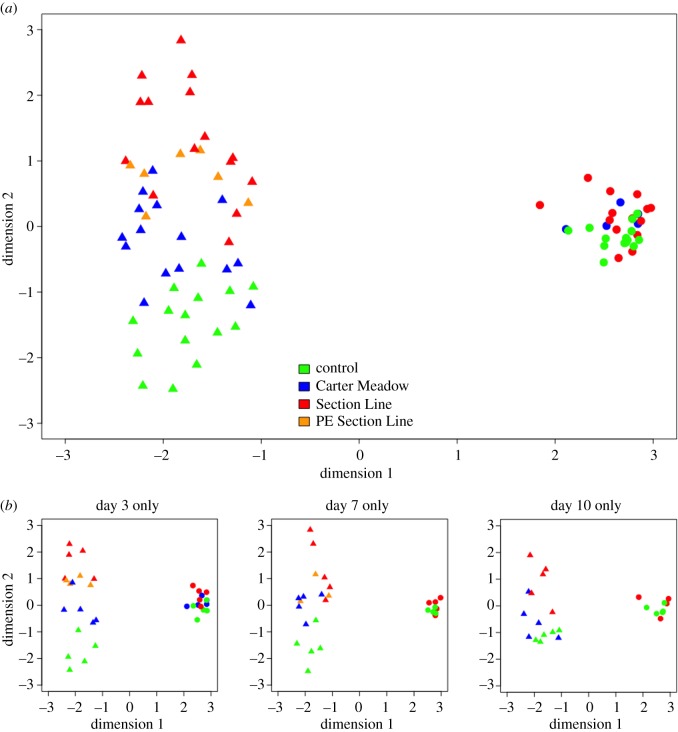
Multidimensional scaling (MDS) plots of 87 RNA-seq samples from an experimental *Bd* exposure study. RNA-seq data shown here represent read counts for 41 646 host-specific contigs (i.e. those from the *Rana clamitans* reference transcriptome). We generated MDS plots using the ‘plotMDS’ function in the R package ‘limma’ [72], specifying visualization of the top 100 contigs that best distinguish samples. All RNA-seq samples are plotted in (*a*), with wood frog samples shown as triangles and American bullfrog samples shown as circles. (*b*) The same data subset by day of tissue harvesting (3, 7 or 10 days post-exposure) to illustrate temporal trends in sample clustering.

After filtering out contigs with low expression, our host-specific RNA-seq dataset was composed of reads mapping to 41 646 contigs. Multidimensional scaling of the RNA-seq data revealed strong sample separation by host species (figure 6). Wood frog samples also showed clustering by treatment in a pattern congruent with frog health throughout the study. For example, control samples clustered most closely with CM *Bd* samples, which came from frogs that had high survival despite *Bd* challenge. SL *Bd* samples, derived from animals experiencing severe chytridiomycosis, were dispersed farthest from controls. There was little separation by treatment among American bullfrog samples.

### 3.6. Host gene expression—expression patterns within species

**Figure 7.**
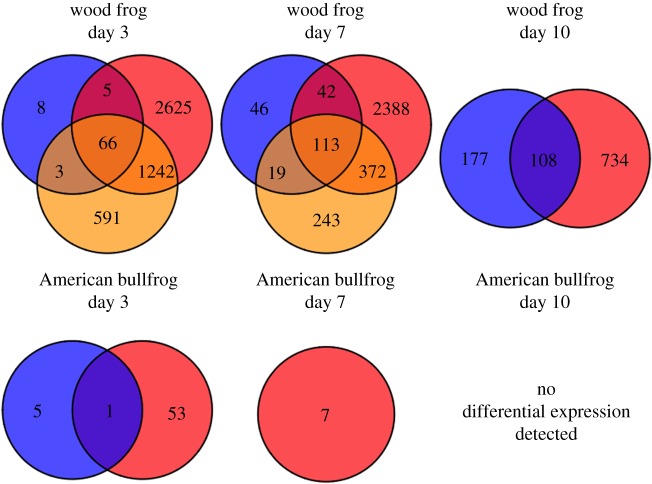
Venn diagrams showing numbers of differentially expressed contigs throughout an experimental *Bd* exposure study. Differential expression analyses were conducted using the R package ‘edgeR’. Here, we compared *Bd* exposure treatment samples within species to time-matched control samples. Venn diagrams show overlap in differentially expressed contig sets within species and time points. CM *Bd* treatment groups are shown as blue circles, SL *Bd* treatments are in red, and orange represents SL *Bd* exposure following previous experimental exposure. There were no CM *Bd* samples for American bullfrogs at day 7 or day 10 post-exposure, and no differential expression was detected between SL *Bd*-exposed and control American bullfrogs at day 10.

**Figure 8.**
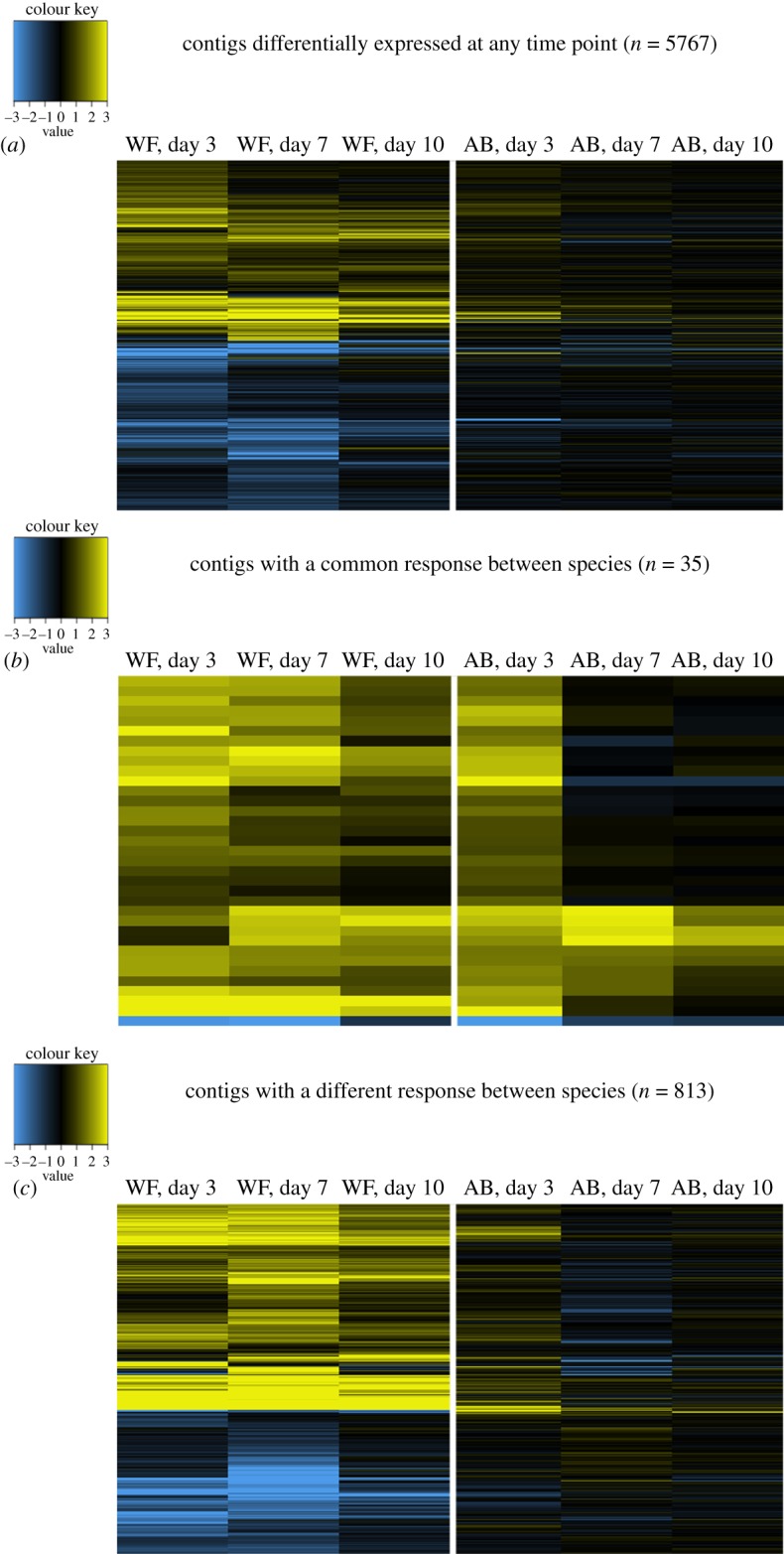
Heatmaps contrasting wood frog and American bullfrog gene expression responses to SL *Bd* exposure. In all panels, each column of the heatmap represents a given amphibian host species (‘WF’ for wood frogs, ‘AB’ for American bullfrogs) at a given tissue sampling time point. Colour within the heatmap represents log2 fold change relative to time-matched control samples, with yellow indicating upregulation and blue indicating downregulation. Contig sets, and thus rows of the heatmap, differ between panels. (*a*) Contigs that were differentially expressed in either host species at any time point. (*b*) A strict subset of contigs represented in (*a*), specifically those that show differential expression in a common direction in both species at any time point. In contrast, (*c*) contigs with a significantly different response to SL *Bd* exposure between the two species at any time point.

**Figure 9.**
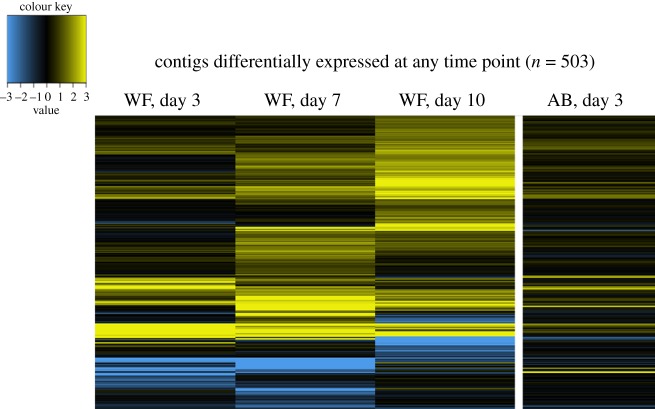
Heatmap contrasting wood frog and American bullfrog gene expression responses to CM *Bd* exposure. Each column of the heatmap represents a given amphibian host species (‘WF’ for wood frogs, ‘AB’ for American bullfrogs) at a given tissue harvesting time point. Colour within the heatmap represents log2 fold change relative to time-matched control samples, yellow indicating upregulation and blue indicating downregulation. Rows of the heatmap represent contigs that were differentially expressed in either host species at any time point. In contrast to SL *Bd* treatments (figure 8), we did not identify any contigs that showed a common or significantly different response to CM *Bd* exposure between amphibian host species.

As suggested by sample clustering on the multidimensional scaling plot, formal differential expression analyses confirmed that *Bd*-exposed wood frogs responded with greater changes in gene expression than *Bd*-exposed American bullfrogs (figures 7, 8*a* and 9). Wood frogs exposed to SL *Bd* had 3938 differentially expressed contigs at 3 days post-exposure. In contrast, American bullfrogs from the same treatment and time point only showed 54 differentially expressed contigs (figure 7). Similar patterns were observed in the SL *Bd* samples collected later in the experiment and in CM *Bd* samples from 3 days post-exposure, with wood frogs having greater numbers of differentially expressed contigs in all cases. In fact, SL *Bd*-exposed American bullfrogs only had seven differentially expressed contigs at 7 days post-exposure and none at day 10, orders of magnitude less differential expression than was observed in the equivalent *Bd*-exposed wood frogs. In wood frogs, differentially expressed contig sets from SL *Bd* treatment groups overlapped more with PE SL *Bd* treatments than with CM *Bd* treatments (figure 7). Generally, differential expression was greatest at earlier sampling time points for both host species (figures 7, 8*a* and 9).

Wood frogs in all *Bd* exposure treatment groups increased expression of contigs associated with antigen processing and presentation (electronic supplementary material, table S4). Upregulation of adaptive immune-related contigs was especially pronounced in the CM *Bd* and PE SL *Bd* treatments. In contrast, American bullfrogs, which had little differential expression overall, did not show immune-associated responses to *Bd* exposure. Contigs differentially expressed in American bullfrogs generally involved metabolic or biosynthesis processes, and American bullfrogs exposed to SL *Bd* showed upregulation of ‘keratinization’ (GO:0031424).

**Figure 10.**
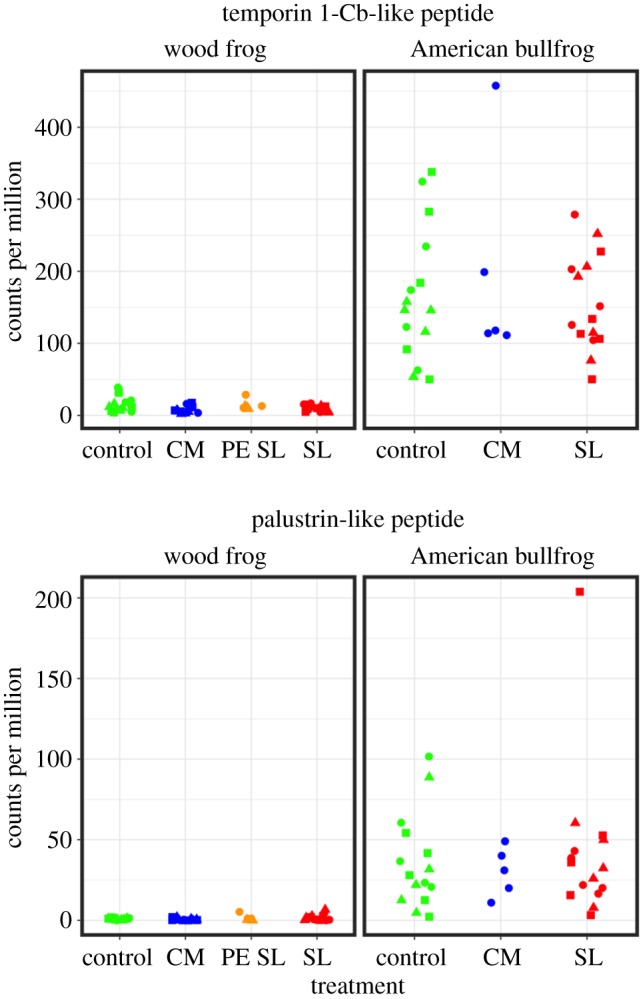
Jitter plots showing read counts for two antimicrobial peptide (AMP) sequences in wood frogs and American bullfrogs. The AMP sequences shown here were labelled Lcla-B and Lcla-H by Robertson & Cornman [63] and share close affinity with temporin 1-Cb and palustrin, respectively. Counts for each sample were scaled to counts per million. Circles represent samples collected 3 days post-exposure, triangles those from day 7, and squares those from day 10. *X*-axis labels represent experimental treatment groups: sham controls, Carter Meadow *Bd* exposure (CM), Section Line *Bd* exposure (SL) and Section Line *Bd* exposure following previous experimental exposure (PE SL).

**Figure 11.**
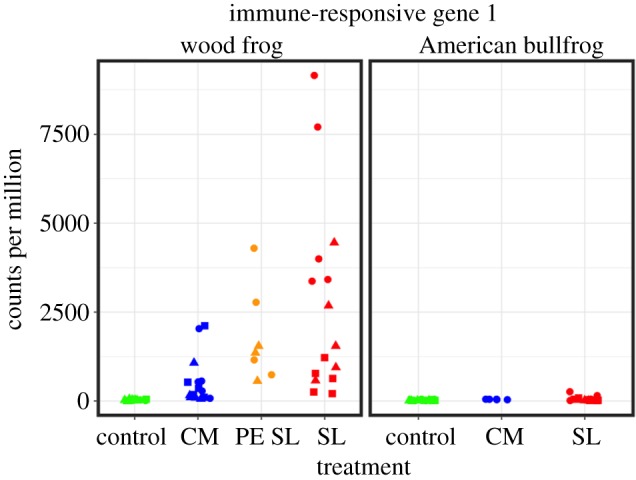
Jitter plot showing read counts for immune-responsive gene 1 (*Irg1*) in wood frogs and American bullfrogs. Data shown here represent reads mapping to *Rana clamitans* contig 252 as assembled by Robertson & Cornman [63]. Counts for each sample were scaled to counts per million. Circles represent samples collected 3 days post-exposure, triangles those from day 7, and squares those from day 10. *X*-axis labels represent experimental treatment groups: sham controls, Carter Meadow *Bd* exposure (CM), Section Line *Bd* exposure (SL), and Section Line *Bd* exposure following previous experimental exposure (PE SL).

Specific contigs with immune or defensive functions showed intriguing expression patterns across host species and treatment groups. For example, seven of 11 AMP contigs were represented in our filtered host-specific RNA-seq dataset. Although these contigs were not differentially expressed in either host species as a result of *Bd* exposure, two AMP sequences were expressed more highly across American bullfrog samples than in wood frogs (figure 10). These AMPs share close affinity with temporin 1C-b and palustrin, respectively. In addition, *R. clamitans* contig 252 was among the 10 most strongly upregulated contigs in wood frogs at three treatment group–time point combinations: CM *Bd*-exposed frogs at both 3 and 10 days post-exposure and SL *Bd*-exposed frogs at 3 days post-exposure (figure 11). This contig was annotated as immune-responsive gene 1 and did not show differential regulation in *Bd*-exposed American bullfrogs.

### 3.7. Host gene expression—comparison of species' responses to *Bd* exposure

**Figure 12.**
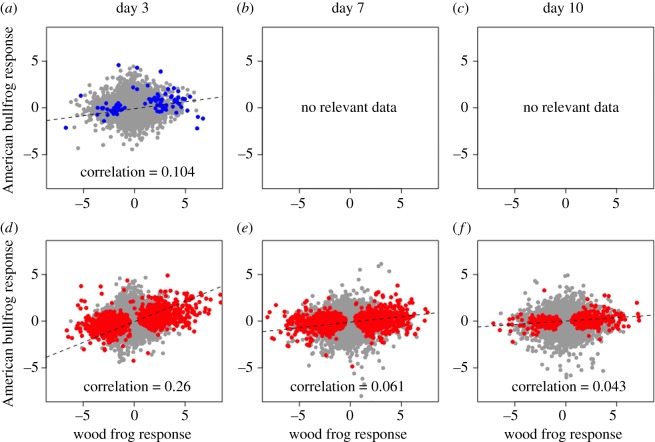
Comparison of species' gene expression responses to *Bd* exposure. (*a*–*c*) Host gene expression responses to CM *Bd* exposure. (*d*–*f*) Responses to SL *Bd*. Axes show log2 fold change between exposed and control frogs. Each point represents one of the 41 646 contigs in our host-specific RNA-seq dataset. Pearson correlation coefficients are given in each plot. Differentially expressed contigs (for either host species) are shown in blue for CM *Bd* treatments and in red for SL *Bd* treatments. There were no CM *Bd* samples for American bullfrogs at day 7 or day 10 post-exposure, precluding comparison of species' responses to *Bd* exposure at those time points for that isolate.

Explicit comparison of wood frog and American bullfrog responses to *Bd* exposure revealed distinct species' responses. First, there was low correlation (*r* ≤ 0.26) in species' responses to *Bd* across *Bd* isolates and time points (figure 12). Correlation of responses was highest between SL *Bd* groups at 3 days post-exposure but declined at later time points. Furthermore, only 35 contigs showed a common response to SL *Bd* in wood frogs and American bullfrogs (figure 8*b*), in contrast to 813 contigs that showed a divergent response between species (figure 8*c*). Contigs showing upregulation in response to SL *Bd* challenge across both species had functional significance for epidermis development and maintenance (table 1). Examination of the contigs that showed different responses to SL *Bd* exposure highlighted molecular processes that were especially distinct between species. In particular, wood frog responses to SL *Bd* exposure were characterized by increased regulation of a variety of immune-related contigs relative to American bullfrogs (table 1). These included contigs annotated with ‘positive regulation of adaptive immune response’ (GO:0002821), ‘positive regulation of leucocyte-mediated immunity’ (GO:0002705), ‘positive regulation of MHC class II biosynthetic process' (GO:0045348), and several innate immune-related processes. Contigs showing significantly downregulated responses in SL *Bd*-exposed wood frogs relative to American bullfrogs included those involved in cell-to-cell adhesion, ion transport and gas exchange (table 1). We did not identify any contigs as having shared or significantly different responses to CM *Bd* exposure between amphibian host species.

**Table 1.** Gene ontology (GO) term enrichment analysis for contigs showing a common or divergent response to SL *Bd* exposure in wood frogs and American bullfrogs. Differential expression analyses were conducted in ‘edgeR’, and GO term enrichment was performed using ‘GOstats’. We compared all *Bd* exposure treatment samples to time-matched controls for differential expression calling. For GO term enrichment analyses, we then pooled contigs within species and treatment groups showing a given response to exposure (i.e. up- or downregulation) at any time point. The first two rows of this table correspond to the contig set represented in figure 8*b*, whereas the last two rows correspond to the contig set represented in figure 8*c*. Select enriched GO terms (up to 10) within these contig sets are shown.

comparison	direction of differential expression	no. of differentially expressed contigs (no. with GO annotation)	no. of enriched GO terms	select enriched GO terms
common responses	up	34 (17)	51	urea cycle; keratinization; epithelium development; wound healing, spreading of epidermal cells; regulation of epidermis development; morphogenesis of an epithelial sheet; response to ischaemia
common responses	down	1 (0)	N/A	N/A
divergent responses	up (i.e. wood frog regulation response is significantly greater than American bullfrogs)	477 (182)	477	positive regulation of adaptive immune response; positive regulation of leucocyte-mediated immunity; positive regulation of T cell-mediated immunity; T cell aggregation; antigen processing and presentation; positive regulation of MHC class II biosynthetic process; positive regulation of innate immune response; cellular response to interleukin-1; cytokine production; natural killer cell activation involved in immune response
divergent responses	down (i.e. wood frog regulation response is significantly less than American bullfrogs)	336 (160)	305	protein localization to adherens junction; desmosome organization; regulation of actin filament-based movement; regulation of action potential; regulation of blood circulation; adherens junction organization; regulation of muscle contraction; respiratory gaseous exchange

### 3.8. *Bd* gene expression

Reads representing *Bd*-derived transcripts were a relatively small portion of our sequencing dataset. Of 8819 *Bd* contigs in our reference transcriptome, only 306 were represented in our pathogen-specific RNA-seq dataset after initial filtering to remove low expression contigs. For *Bd* gene expression analyses, we further narrowed this dataset to 106 *Bd* contigs that were characterized by low expression across all control samples. Most *Bd* differential expression was observed in wood frog samples collected 3 days post-exposure (electronic supplementary material, table S5). At this time point in this host species, SL *Bd* versus CM *Bd* and PE SL *Bd* versus SL *Bd* comparisons showed 17 and 14 differentially expressed contigs, respectively. Of the 14 contigs downregulated in PE SL *Bd* relative to SL *Bd* at 3 days post-exposure, 13 of these were upregulated in SL *Bd* relative to CM *Bd* at the same time point (electronic supplementary material, table S5). Therefore, these sequences were consistently recovered in greater abundance in samples from naive wood frogs exposed to SL *Bd* than in CM *Bd* or PE SL *Bd* samples. Functions of these contigs included metabolic processes, biosynthesis and transmembrane transport (electronic supplementary material, table S6). We did not detect differential expression in *Bd* contigs recovered from American bullfrog samples.

## 4. Discussion

Our experimental *Bd* exposures resulted in large differences in survival, body condition, infection status and gene expression according to amphibian host species and *Bd* isolate. Wood frogs exposed to SL *Bd* had high infection loads that resulted in drastically reduced survival. In contrast, wood frogs exposed to CM *Bd* had low infection loads and no mortality. American bullfrogs, even those exposed to the especially virulent SL *Bd*, had rapid infection clearance or avoided infection entirely and did not develop disease. While infection dynamics and mortality effects were thus host- and isolate-specific, both host species showed evidence of sublethal effects of *Bd* exposure. For all *Bd* treatments, individuals had significant reduction in body mass in the days immediately after *Bd* challenge. These results suggest there can be costs to *Bd* infection even in resistant or tolerant host species like the American bullfrog [44,52]. Finally, gene expression, our key experimental outcome, also differed considerably between study species and *Bd* isolate treatments. Therefore, distinct molecular responses underlie chytridiomycosis disease progression, or lack thereof, in these two frog species.

While we made every attempt to design and implement a rigorous experimental study, we recognize some underlying assumptions and caveats that should inform interpretation of our results. First, our designation of American bullfrogs as a model ‘*Bd*-resistant species’ may simplify a complex disease response phenotype. Some wild American bullfrog populations have relatively high *Bd* prevalence and load [40,79], and one experimental study suggested that the species can develop chytridiomycosis [80]. Our results here, however, reinforce a pattern established in previous work wherein American bullfrogs were observed to readily resist *Bd* infection or suffer only relatively mild infections [39,41]. We based our initial assumption that American bullfrogs would largely resist *Bd* on these findings. Second, it is possible that not all of our study animals were truly *Bd*-naive. Wood frogs were collected as egg masses from the wild and hatched in controlled laboratory conditions. Thus, it is extremely unlikely they would have been subject to *Bd* exposure prior to experimentation. However, American bullfrogs were collected from the wild at the tadpole stage. This was a logistical necessity stemming from the extended larval period of this species [48], which precluded the time commitment necessary to rear individuals from eggs. Although we visually screened American bullfrog tadpoles for chytridiomycosis in the field [49] and further confirmed all study animals of both species to be *Bd*-negative via qPCR prior to experimentation, it remains possible that some study subjects had a prior history of *Bd* exposure that went undetected by our procedures. Third, numerous characters relevant to *Bd* response, including immunogenetic traits, can vary among amphibian populations within species [32,34,81,82]. Indeed, the potential variation in American bullfrog response to *Bd* mentioned above may be just such a population-specific phenotypic characteristic [39–41,79,80]. Therefore, experimental results on our two study species may be partially driven by particularities of the intraspecific variation represented at our collection localities. Certainly, more work comparing inter- and intraspecific variation in amphibian response to *Bd* is needed to characterize the relative magnitude of these influences. Generally, however, gene expression is expected to reflect phylogenetic relatedness, with populations within species showing greater similarity than comparisons among species [83,84].

Turning to our gene expression results, when comparing between *Bd* isolates, responses of exposed wood frogs provide evidence for varying disease severity and defence strategies. SL *Bd­*-exposed wood frogs had much greater gene expression change overall compared to CM *Bd*-exposed animals, reflecting greater overall morbidity and mortality in this treatment group. Interestingly, all *Bd*-exposed wood frog treatment groups showed upregulation of adaptive immune system components, suggesting these frogs were beginning to mount an antigen-specific immune defence. Furthermore, explicit comparison of species' responses to *Bd* exposure revealed that adaptive and innate immune-related contigs were among those responding most differently between species, with greater expression in wood frogs relative to American bullfrogs. Therefore, immune system activation is a characteristic distinguishing wood frog *Bd* exposure responses from those of American bullfrogs. These results contrast with early studies on amphibian gene expression following *Bd* exposure that suggested a largely absent immune response [28,29] yet support more recent work that shows adaptive immune activation even, or especially, in species that succumb to chytridiomycosis [25,65]. Wood frogs exposed to SL *Bd* also differed from American bullfrogs in that they showed downregulation of various contigs related to skin integrity, including those annotated with the GO terms ‘desmosome organization’ (GO:0002934) and ‘adherens junction organization’ (GO:0034332), indicating cell adhesion functions. These findings are congruent with, and help generalize, previous studies indicating that resistant amphibian hosts tend to upregulate skin integrity pathways in response to *Bd* exposure, whereas in susceptible species these same pathways are often downregulated [25,85,86].

In our study, immune responses were especially apparent in PE SL *Bd* wood frogs, which had multiple terms related to both innate and adaptive immune function in their top enriched GO terms for upregulated contigs, adding to previous evidence that amphibians may develop immunological memory against *Bd* as a consequence of prior exposure [25,31]. However, even apparently primed defences were unable to prevent host death in this case. These trends in immune gene expression also reflect the unique patterns of gene expression we observed in the PE SL *Bd* treatment group more broadly. Although these animals shared a large proportion of differentially expressed contigs with the naive SL *Bd* individuals, they also had a unique response to exposure that represented hundreds of differentially expressed contigs. This stands in contrast to Ellison *et al.* [65], who found little evidence for differences in gene expression response between naive and previously exposed *Atelopus* frogs.

Whether adaptive immune responses were mounted by naive or experienced individuals, why were they apparently ineffective in defending wood frogs against SL *Bd* and preventing host mortality? First, increasing evidence suggests *Bd* actively inhibits essential components of the amphibian adaptive immune system [35–37], potentially rendering it ineffective. Second, immune responses can themselves be damaging to the host, as when widespread inflammation results in immunopathology [87]. Finally, immune defences are costly in general [88,89] and within the amphibian–*Bd* system specifically [51,52]. Therefore, immune activation can necessitate critical trade-offs with other organismal processes [90]. Compared to innate immune defences, trade-offs associated with adaptive immune responses may be especially pronounced [88]. Indeed, wood frogs exposed to *Bd* isolates showed downregulation of genes associated with GO terms including ‘water transport’ (GO:0006833), ‘locomotory behaviour’ (GO:0007626), ‘entrainment of circadian clock by photoperiod’ (GO:0043153), and ‘tissue development’ (GO:0009888), suggesting disruption of various organismal systems as a result of pathogen exposure and associated host responses. Concomitantly, CM *Bd* and PE SL *Bd* wood frogs had upregulation of the GO term ‘cellular response to glucocorticoid stimulus’ (GO:0071385). Glucocorticoid hormones regulate organismal stress responses and have been linked to chytridiomycosis pathogenesis [91,92], while increased activation of stress-related pathways may be a result of *Bd* exposure generally [28,86]. In sum, these results suggest *Bd*-exposed wood frogs manifested substantial physiological stress, which may be partly a consequence of mounting an adaptive immune response. Such costs are also reflected in the significant losses of body mass observed in these animals. The costs of immune system activation are likely to be especially detrimental in cases where they are not balanced by the benefits of effective host defence, as is often the case with chytridiomycosis. In these situations, immune responses may instead result in immunopathology and increased metabolic expenditure at the expense of maintaining other important homeostatic cellular functions.

Interestingly, wood frogs exposed to *Bd* had increased expression of the frog homologue of immune-responsive gene 1 (*Irg1*). *Irg1* was upregulated in every *Bd* exposure treatment group relative to controls, and it seems especially relevant to wood frog response to CM *Bd* given that it was among the top 10 upregulated contigs in this exposure treatment at both the 3 and 10 day time points. Only recently has the mechanistic link between *Irg1* and immune function been elucidated. The gene, which is commonly upregulated as a result of pathogen threat, codes for an enzyme that catalyses the conversion of *cis*-aconitate into itaconic acid [93]. In turn, itaconic acid inhibits a key step of the glyoxylate shunt, a metabolic pathway used by various microbes under certain environmental conditions; this inhibition accounts for itaconic acid's antimicrobial activity [93,94]. Critically, the glyoxylate shunt pathway contributes to virulence of fungal pathogens in general [95] and may be linked to *Bd* growth specifically [96]. Furthermore, *Irg1* is upregulated in macrophages following pathogen challenge [93], and macrophages are among the innate immune cell types that are unaffected by the *Bd* inhibitory factors that disrupt the amphibian adaptive immune system [35]. Thus, *Irg1* and itaconic acid deserve further attention as a feasible mechanism contributing to amphibian host control of *Bd* infection.

In contrast to wood frogs, American bullfrogs avoided chytridiomycosis without major changes in gene expression. The highly similar gene expression profiles of control and *Bd*-exposed American bullfrogs (figures 6–9), in conjunction with qPCR and histology results that showed generally low *Bd* loads or absence of infection, demonstrate that this species responds rapidly and effectively to limit *Bd* invasion with minimal disruption of normal physiology. These results all suggest American bullfrogs use constitutively active defences to combat *Bd*. Skin morphological characteristics are a good candidate for such a defence [16,18,86,97,98]. Specifically, American bullfrogs are known to slough rapidly following *Bd* exposure and have a thickened epidermis relative to wood frogs [18,99]. Skin sloughing can be an effective mechanism for reducing microbial abundance on the epidermis [19], and other amphibians are able to clear *Bd* infections through sloughing [20]. If the particular morphological characteristics of American bullfrogs allow for an increased rate or extent of skin sloughing, then these traits may explain the species' ability to rapidly clear *Bd* infection and resist chytridiomycosis [39,41].

Alternatively, other constitutively active skin-associated defences could account for American bullfrog avoidance of chytridiomycosis. We found evidence that at least two AMPs, belonging to the temporin and palustrin families, respectively, were highly expressed in American bullfrogs relative to wood frogs. These results are unsurprising given that AMP communities are highly species-specific [100]. American bullfrogs have a robust suite of at least 15 known AMPs [22,101,102], whereas wood frogs appear to produce only a single AMP [103,104]. In addition, AMP production can be environmentally influenced [103], and thus wood frogs might have had increased AMP defences under more favourable experimental conditions (e.g. increased temperature [105]). Finally, although all of our frogs were reared in a common environment, species-specific skin-associated microbes could represent another component of American bullfrog defences against *Bd* [106]. Amphibian skin can strongly filter environmental microbes [107], and thus the skin microbiota on American bullfrogs may have differed significantly from wood frogs at the time of *Bd* exposure despite the two species sharing a similar rearing environment. Although little work has directly examined the interactions between American bullfrog microbial communities and *Bd*, American bullfrog-associated bacterial species in the families Bacillaceae and Xanthomonadaceae deserve more attention for their potential role in host defence given their ability to inhibit dermatophyte fungi [108].

Despite great differences in realized virulence on amphibian hosts, our analyses showed little difference in gene expression between CM *Bd* and SL *Bd*. Virulence in *Bd* may be driven in part by the pathogen's basic growth rate. Previous work has reported more rapid growth of SL *Bd* than other isolates on amphibian hosts [45], which agrees with our results showing high infection loads in SL *Bd*-exposed frogs. We observed anecdotally that SL *Bd* grew more rapidly in culture than CM *Bd*, and prior data show that SL *Bd* also grows more densely at carrying capacity [45]. Virulence of SL and CM *Bd* isolates may, therefore, be driven by differences in the speed and severity of *Bd* invasion and resulting disruption of host tissues.

In conclusion, our two study species mount idiosyncratic defences to *Bd* threat, one successfully and the other far less so. Wood frogs employ adaptive immune responses that may defend against CM *Bd* but are clearly insufficient to prevent death after exposure to SL *Bd*. In addition, pathways associated with innate immune responses, such as *Irg1*-mediated catalysis of itaconic acid, may provide further defence from *Bd*. In contrast, American bullfrogs show little evidence of an adaptive immune response, yet they have better health outcomes after *Bd* exposure. Other recent work also found limited gene expression responses to *Bd* exposure in resistant amphibian hosts [85,86]. For example, in a comparative study of four species, Ellison *et al.* [85] found that gene expression of the most resistant amphibian tested, *Agalychnis callidryas*, was relatively unresponsive to *Bd* exposure. Similarly, Poorten & Rosenblum [86] evaluated a pair of bufonid species expected to differ in *Bd* susceptibility and showed that the more resistant congener had a much weaker transcriptional response to pathogen challenge. Thus, our results bolster the idea that a muted gene expression response may broadly characterize species that are not susceptible to chytridiomycosis. American bullfrog defences likely consist of a combination of species-specific skin morphological properties and skin-associated innate immune components. The present comparative study shows that while adaptive immune responses to *Bd* exist, they are not sufficient to ensure survival in some species and may be unnecessary in others. Our findings move us closer to understanding the complex system of defence mechanisms that can help amphibian species cope with the significant global threat posed by *Bd*.

## Supplementary Material

Supplementary material for “Gene expression differs in susceptible and resistant amphibians exposed to Batrachochytrium dendrobatidis”

## Data accessibility

Raw RNA sequencing data: NCBI Sequence Read Archive accession number SRP111327. Further data and code supporting all analyses are available at https://github.com/eveskew/frog_chytrid_transcriptomics.
