# Supplementary material for: Gene expression differs in susceptible and resistant amphibians exposed to Batrachochytrium dendrobatidis
Source: R Soc Open Sci. 2018 Feb 28;5(2):170910. doi: 10.1098/rsos.170910 (PMC5830717; doi:10.1098/rsos.170910)
Supplement: Supplementary material for “Gene expression differs in susceptible and resistant amphibians exposed to Batrachochytrium dendrobatidis” [file rsos170910supp1.docx]

Electronic Supplementary Material

**Gene expression differs in susceptible and resistant amphibians exposed to *Batrachochytrium dendrobatidis***

Evan A. Eskew^1,2^, Barbara C. Shock^3,4^, Elise E. B. LaDouceur^5^, Kevin Keel^4^, Michael R. Miller^6^, Janet E. Foley^7^, Brian D. Todd^8^

^1^ Graduate Group in Ecology, University of California, Davis, Davis CA

^2^ EcoHealth Alliance, New York NY

^3^ Department of Biology, Lincoln Memorial University, Harrogate TN

^4^ Department of Pathology, Microbiology, and Immunology, School of Veterinary Medicine, University of California, Davis, Davis CA

^5^ Northwest ZooPath, Monroe WA

^6^ Department of Animal Science, University of California, Davis, Davis CA

^7^ Department of Medicine and Epidemiology, School of Veterinary Medicine, University of California, Davis, Davis CA

^8^ Department of Wildlife, Fish, and Conservation Biology, University of California, Davis, Davis CA

Author for correspondence: Evan A. Eskew; email: eveskew@gmail.com

**Table S1.** Sample sizes across amphibian host species, experimental treatment groups, and time points (days post-exposure) for three different study measurements. These data are intended to clarify sample sizes for analyses presented in the main text, figures 1−4. The measurements in question, their sample sizes, and the relevant main text figures are given in their respective columns. Sample sizes may differ over time within treatment groups because of animal removal for tissue harvesting (destructive sampling) or because of disease-related morbidity. In addition, for swab sampling and subsequent qPCR analyses, random subsampling (rather than complete sampling) occurred within control groups since these animals were all expected to test negative for *Bd*. Notes highlighting these issues are included where relevant.

| **Species** | **Treatment** | **Days Post-Exposure** | **Measurement** | **Sample Size** | **Relevant Figures** | **Notes** |
| --- | --- | --- | --- | --- | --- | --- |
| Wood Frog | Control | 0 | Initial Sample Size | 35 | 1, 2 |  |
| Wood Frog | Carter Meadow | 0 | Initial Sample Size | 35 | 1, 2 |  |
| Wood Frog | Section Line | 0 | Initial Sample Size | 35 | 1, 2 |  |
| Wood Frog | PE Section Line | 0 | Initial Sample Size | 8 | 1, 2 |  |
| American Bullfrog | Control | 0 | Initial Sample Size | 35 | 1, 2 |  |
| American Bullfrog | Carter Meadow | 0 | Initial Sample Size | 5 | 1, 2 |  |
| American Bullfrog | Section Line | 0 | Initial Sample Size | 35 | 1, 2 |  |
|  |  |  |  |  |  |  |
| Wood Frog | Control | 4 | Infection Prevalence | 10 | 3 | Random subsampling of surviving individuals |
| Wood Frog | Control | 11 | Infection Prevalence | 10 | 3 | Random subsampling of surviving individuals |
| Wood Frog | Control | 18 | Infection Prevalence | 10 | 3 | Random subsampling of surviving individuals |
| Wood Frog | Control | 25 | Infection Prevalence | 5 | 3 | Random subsampling of surviving individuals |
| Wood Frog | Control | 32 | Infection Prevalence | 5 | 3 | Random subsampling of surviving individuals |
| Wood Frog | Control | 39 | Infection Prevalence | 5 | 3 | Random subsampling of surviving individuals |
| Wood Frog | Control | 46 | Infection Prevalence | 5 | 3 | Random subsampling of surviving individuals |
|  |  |  |  |  |  |  |
| Wood Frog | Carter Meadow | 4 | Infection Prevalence | 30 | 3 | Complete sampling of surviving individuals |
| Wood Frog | Carter Meadow | 11 | Infection Prevalence | 20 | 3 | Complete sampling of surviving individuals |
| Wood Frog | Carter Meadow | 18 | Infection Prevalence | 20 | 3 | Complete sampling of surviving individuals |
| Wood Frog | Carter Meadow | 25 | Infection Prevalence | 20 | 3 | Complete sampling of surviving individuals |
| Wood Frog | Carter Meadow | 32 | Infection Prevalence | 20 | 3 | Complete sampling of surviving individuals |
| Wood Frog | Carter Meadow | 39 | Infection Prevalence | 20 | 3 | Complete sampling of surviving individuals |
| Wood Frog | Carter Meadow | 46 | Infection Prevalence | 20 | 3 | Complete sampling of surviving individuals |
|  |  |  |  |  |  |  |
| Wood Frog | Section Line | 4 | Infection Prevalence | 30 | 3 | Complete sampling of surviving individuals |
| Wood Frog | Section Line | 11 | Infection Prevalence | 2 | 3 | Complete sampling of surviving individuals |
| Wood Frog | Section Line | 18 | Infection Prevalence | 1 | 3 | Complete sampling of surviving individuals |
| Wood Frog | Section Line | 25 | Infection Prevalence | 1 | 3 | Complete sampling of surviving individuals |
| Wood Frog | Section Line | 32 | Infection Prevalence | 1 | 3 | Complete sampling of surviving individuals |
| Wood Frog | Section Line | 39 | Infection Prevalence | 1 | 3 | Complete sampling of surviving individuals |
| Wood Frog | Section Line | 46 | Infection Prevalence | 1 | 3 | Complete sampling of surviving individuals |
|  |  |  |  |  |  |  |
| Wood Frog | PE Section Line | 4 | Infection Prevalence | 4 | 3 | Complete sampling of surviving individuals |
| Wood Frog | PE Section Line | 11 | Infection Prevalence | N/A | 3 | No surviving individuals in treatment |
| Wood Frog | PE Section Line | 18 | Infection Prevalence | N/A | 3 | No surviving individuals in treatment |
| Wood Frog | PE Section Line | 25 | Infection Prevalence | N/A | 3 | No surviving individuals in treatment |
| Wood Frog | PE Section Line | 32 | Infection Prevalence | N/A | 3 | No surviving individuals in treatment |
| Wood Frog | PE Section Line | 39 | Infection Prevalence | N/A | 3 | No surviving individuals in treatment |
| Wood Frog | PE Section Line | 46 | Infection Prevalence | N/A | 3 | No surviving individuals in treatment |
|  |  |  |  |  |  |  |
| American Bullfrog | Control | 4 | Infection Prevalence | 10 | 3 | Random subsampling of surviving individuals |
| American Bullfrog | Control | 11 | Infection Prevalence | 10 | 3 | Random subsampling of surviving individuals |
| American Bullfrog | Control | 18 | Infection Prevalence | 10 | 3 | Random subsampling of surviving individuals |
| American Bullfrog | Control | 25 | Infection Prevalence | 5 | 3 | Random subsampling of surviving individuals |
| American Bullfrog | Control | 32 | Infection Prevalence | 5 | 3 | Random subsampling of surviving individuals |
| American Bullfrog | Control | 39 | Infection Prevalence | 5 | 3 | Random subsampling of surviving individuals |
| American Bullfrog | Control | 46 | Infection Prevalence | 5 | 3 | Random subsampling of surviving individuals |
|  |  |  |  |  |  |  |
| American Bullfrog | Carter Meadow | 4 | Infection Prevalence | N/A | 3 | No surviving individuals in treatment |
| American Bullfrog | Carter Meadow | 11 | Infection Prevalence | N/A | 3 | No surviving individuals in treatment |
| American Bullfrog | Carter Meadow | 18 | Infection Prevalence | N/A | 3 | No surviving individuals in treatment |
| American Bullfrog | Carter Meadow | 25 | Infection Prevalence | N/A | 3 | No surviving individuals in treatment |
| American Bullfrog | Carter Meadow | 32 | Infection Prevalence | N/A | 3 | No surviving individuals in treatment |
| American Bullfrog | Carter Meadow | 39 | Infection Prevalence | N/A | 3 | No surviving individuals in treatment |
| American Bullfrog | Carter Meadow | 46 | Infection Prevalence | N/A | 3 | No surviving individuals in treatment |
|  |  |  |  |  |  |  |
| American Bullfrog | Section Line | 4 | Infection Prevalence | 30 | 3 | Complete sampling of surviving individuals |
| American Bullfrog | Section Line | 11 | Infection Prevalence | 20 | 3 | Complete sampling of surviving individuals |
| American Bullfrog | Section Line | 18 | Infection Prevalence | 19 | 3 | Complete sampling of surviving individuals |
| American Bullfrog | Section Line | 25 | Infection Prevalence | 19 | 3 | Complete sampling of surviving individuals |
| American Bullfrog | Section Line | 32 | Infection Prevalence | 19 | 3 | Complete sampling of surviving individuals |
| American Bullfrog | Section Line | 39 | Infection Prevalence | 19 | 3 | Complete sampling of surviving individuals |
| American Bullfrog | Section Line | 46 | Infection Prevalence | 19 | 3 | Complete sampling of surviving individuals |
|  |  |  |  |  |  |  |
| Wood Frog | Control | 4 | Infection Load  (of Positive Individuals) | N/A | 4 | No positive individuals in treatment |
| Wood Frog | Control | 11 | Infection Load  (of Positive Individuals) | N/A | 4 | No positive individuals in treatment |
| Wood Frog | Control | 18 | Infection Load  (of Positive Individuals) | N/A | 4 | No positive individuals in treatment |
| Wood Frog | Control | 25 | Infection Load  (of Positive Individuals) | N/A | 4 | No positive individuals in treatment |
| Wood Frog | Control | 32 | Infection Load  (of Positive Individuals) | N/A | 4 | No positive individuals in treatment |
| Wood Frog | Control | 39 | Infection Load  (of Positive Individuals) | N/A | 4 | No positive individuals in treatment |
| Wood Frog | Control | 46 | Infection Load  (of Positive Individuals) | N/A | 4 | No positive individuals in treatment |
|  |  |  |  |  |  |  |
| Wood Frog | Carter Meadow | 4 | Infection Load  (of Positive Individuals) | 21 | 4 |  |
| Wood Frog | Carter Meadow | 11 | Infection Load  (of Positive Individuals) | 17 | 4 |  |
| Wood Frog | Carter Meadow | 18 | Infection Load  (of Positive Individuals) | 14 | 4 |  |
| Wood Frog | Carter Meadow | 25 | Infection Load  (of Positive Individuals) | 15 | 4 |  |
| Wood Frog | Carter Meadow | 32 | Infection Load  (of Positive Individuals) | 14 | 4 |  |
| Wood Frog | Carter Meadow | 39 | Infection Load  (of Positive Individuals) | 16 | 4 |  |
| Wood Frog | Carter Meadow | 46 | Infection Load  (of Positive Individuals) | 14 | 4 |  |
|  |  |  |  |  |  |  |
| Wood Frog | Section Line | 4 | Infection Load  (of Positive Individuals) | 30 | 4 |  |
| Wood Frog | Section Line | 11 | Infection Load  (of Positive Individuals) | 2 | 4 |  |
| Wood Frog | Section Line | 18 | Infection Load  (of Positive Individuals) | N/A | 4 | No positive individuals in treatment |
| Wood Frog | Section Line | 25 | Infection Load  (of Positive Individuals) | N/A | 4 | No positive individuals in treatment |
| Wood Frog | Section Line | 32 | Infection Load  (of Positive Individuals) | N/A | 4 | No positive individuals in treatment |
| Wood Frog | Section Line | 39 | Infection Load  (of Positive Individuals) | N/A | 4 | No positive individuals in treatment |
| Wood Frog | Section Line | 46 | Infection Load  (of Positive Individuals) | 1 | 4 |  |
|  |  |  |  |  |  |  |
| Wood Frog | PE Section Line | 4 | Infection Load  (of Positive Individuals) | 4 | 4 |  |
| Wood Frog | PE Section Line | 11 | Infection Load  (of Positive Individuals) | N/A | 4 | No surviving individuals in treatment |
| Wood Frog | PE Section Line | 18 | Infection Load  (of Positive Individuals) | N/A | 4 | No surviving individuals in treatment |
| Wood Frog | PE Section Line | 25 | Infection Load  (of Positive Individuals) | N/A | 4 | No surviving individuals in treatment |
| Wood Frog | PE Section Line | 32 | Infection Load  (of Positive Individuals) | N/A | 4 | No surviving individuals in treatment |
| Wood Frog | PE Section Line | 39 | Infection Load  (of Positive Individuals) | N/A | 4 | No surviving individuals in treatment |
| Wood Frog | PE Section Line | 46 | Infection Load  (of Positive Individuals) | N/A | 4 | No surviving individuals in treatment |
|  |  |  |  |  |  |  |
| American Bullfrog | Control | 4 | Infection Load  (of Positive Individuals) | N/A | 4 | No positive individuals in treatment |
| American Bullfrog | Control | 11 | Infection Load  (of Positive Individuals) | N/A | 4 | No positive individuals in treatment |
| American Bullfrog | Control | 18 | Infection Load  (of Positive Individuals) | N/A | 4 | No positive individuals in treatment |
| American Bullfrog | Control | 25 | Infection Load  (of Positive Individuals) | N/A | 4 | No positive individuals in treatment |
| American Bullfrog | Control | 32 | Infection Load  (of Positive Individuals) | N/A | 4 | No positive individuals in treatment |
| American Bullfrog | Control | 39 | Infection Load  (of Positive Individuals) | N/A | 4 | No positive individuals in treatment |
| American Bullfrog | Control | 46 | Infection Load  (of Positive Individuals) | N/A | 4 | No positive individuals in treatment |
|  |  |  |  |  |  |  |
| American Bullfrog | Carter Meadow | 4 | Infection Load  (of Positive Individuals) | N/A | 4 | No surviving individuals in treatment |
| American Bullfrog | Carter Meadow | 11 | Infection Load  (of Positive Individuals) | N/A | 4 | No surviving individuals in treatment |
| American Bullfrog | Carter Meadow | 18 | Infection Load  (of Positive Individuals) | N/A | 4 | No surviving individuals in treatment |
| American Bullfrog | Carter Meadow | 25 | Infection Load  (of Positive Individuals) | N/A | 4 | No surviving individuals in treatment |
| American Bullfrog | Carter Meadow | 32 | Infection Load  (of Positive Individuals) | N/A | 4 | No surviving individuals in treatment |
| American Bullfrog | Carter Meadow | 39 | Infection Load  (of Positive Individuals) | N/A | 4 | No surviving individuals in treatment |
| American Bullfrog | Carter Meadow | 46 | Infection Load  (of Positive Individuals) | N/A | 4 | No surviving individuals in treatment |
|  |  |  |  |  |  |  |
| American Bullfrog | Section Line | 4 | Infection Load  (of Positive Individuals) | 15 | 4 |  |
| American Bullfrog | Section Line | 11 | Infection Load  (of Positive Individuals) | 3 | 4 |  |
| American Bullfrog | Section Line | 18 | Infection Load  (of Positive Individuals) | N/A | 4 | No positive individuals in treatment |
| American Bullfrog | Section Line | 25 | Infection Load  (of Positive Individuals) | N/A | 4 | No positive individuals in treatment |
| American Bullfrog | Section Line | 32 | Infection Load  (of Positive Individuals) | N/A | 4 | No positive individuals in treatment |
| American Bullfrog | Section Line | 39 | Infection Load  (of Positive Individuals) | 1 | 4 |  |
| American Bullfrog | Section Line | 46 | Infection Load  (of Positive Individuals) | N/A | 4 | No positive individuals in treatment |

**Table S2.** Sample sizes for RNA-seq analyses across amphibian host species, experimental treatment groups, and time points (days post-exposure). Most conditions are represented by five biological replicates. Previously exposed (PE) Section Line *Bd* wood frogs have smaller sample sizes because of the smaller initial number of animals in that treatment group. Carter Meadow *Bd*-exposed American bullfrogs were not available past day 3 post-exposure because insufficient quantities of that *Bd* isolate did not allow for initial exposure of a larger sample of American bullfrogs. In total, 87 RNA-seq samples were analyzed.

|  |  | Days Post-Exposure | | |
| --- | --- | --- | --- | --- |
|  |  | **3** | **7** | **10** |
| Species | Treatment |  |  |  |
| **Wood Frog** | **Control** | 5 | 5 | 5 |
|  | **Carter Meadow** | 5 | 5 | 5 |
|  | **Section Line** | 5 | 5 | 5 |
|  | **PE Section Line** | 4 | 3 | N/A |
| **American Bullfrog** | **Control** | 5 | 5 | 5 |
|  | **Carter Meadow** | 5 | N/A | N/A |
|  | **Section Line** | 5 | 5 | 5 |

**Table S3.** Histology infection scores and *Bd* infection loads via quantitative PCR for 90 frogs. Histology infection scores represent a measure of infection load, and possible scores ranged from 0–300. *Bd* infection load was also evaluated with a quantitative PCR (qPCR) assay, using frog skin swab samples as starting material. See the main text for further detail on both of these infection detection methods. For each individual, *Bd* infection load via qPCR represents data derived from the swab sample collected closest to the time of euthanasia (i.e., Days Post-Exposure). Within species and treatment, samples are sorted by increasing histology infection score.

| **Species** | **ID** | **Treatment** | **Days**  **Post-Exposure** | **Sacrificed**  **For Tissue Harvest?** | **Histology Infection Score** | ***Bd* Load**  **via qPCR** |
| --- | --- | --- | --- | --- | --- | --- |
| American Bullfrog | 11 | Control | 7 | Y | 0 | 0 |
| American Bullfrog | 15 | Control | 10 | Y | 0 | 0 |
| American Bullfrog | 43 | Control | 10 | Y | 0 | 0 |
| American Bullfrog | 44 | Control | 10 | Y | 0 | 0 |
| American Bullfrog | 53 | Control | 7 | Y | 0 | 0 |
| American Bullfrog | 68 | Control | 10 | Y | 0 | 0 |
| American Bullfrog | 73 | Control | 22 | N | 0 | 0 |
| American Bullfrog | 86 | Control | 7 | Y | 0 | 0 |
| American Bullfrog | 87 | Control | 10 | Y | 0 | 0 |
| American Bullfrog | 101 | Control | 7 | Y | 0 | 0 |
| American Bullfrog | 111 | Control | 7 | Y | 0 | 0 |
| American Bullfrog | 9 | Carter Meadow | 3 | Y | 0 | 36.80 |
| American Bullfrog | 30 | Carter Meadow | 3 | Y | 0 | 131.20 |
| American Bullfrog | 36 | Carter Meadow | 3 | Y | 0 | 427.20 |
| American Bullfrog | 60 | Carter Meadow | 3 | Y | 2 | 44.80 |
| American Bullfrog | 83 | Carter Meadow | 3 | Y | 2 | 1128.00 |
| American Bullfrog | 37 | Section Line | 10 | Y | 0 | 0 |
| American Bullfrog | 46 | Section Line | 10 | Y | 0 | 0 |
| American Bullfrog | 62 | Section Line | 11 | N | 0 | 0 |
| American Bullfrog | 64 | Section Line | 10 | Y | 0 | 12.27 |
| American Bullfrog | 79 | Section Line | 7 | Y | 0 | 0 |
| American Bullfrog | 81 | Section Line | 7 | Y | 0 | 0 |
| American Bullfrog | 84 | Section Line | 7 | Y | 0 | 0 |
| American Bullfrog | 85 | Section Line | 7 | Y | 0 | 0 |
| American Bullfrog | 93 | Section Line | 10 | Y | 0 | 0 |
| American Bullfrog | 96 | Section Line | 7 | Y | 0 | 72.00 |
| American Bullfrog | 59 | Untreated | NA | N | 0 | 0 |
| Wood Frog | 2 | Control | 10 | Y | 0 | 0 |
| Wood Frog | 14 | Control | 3 | Y | 0 | 0 |
| Wood Frog | 15 | Control | 7 | Y | 0 | 0 |
| Wood Frog | 18 | Control | 3 | Y | 0 | 0 |
| Wood Frog | 23 | Control | 10 | Y | 0 | 360.00 |
| Wood Frog | 50 | Control | 10 | Y | 0 | 126.93 |
| Wood Frog | 51 | Control | 7 | Y | 0 | 0 |
| Wood Frog | 74 | Control | 7 | Y | 0 | 0 |
| Wood Frog | 90 | Control | 10 | Y | 0 | 0 |
| Wood Frog | 108 | Control | 7 | Y | 0 | 0 |
| Wood Frog | 142 | Control | 10 | Y | 0 | 0 |
| Wood Frog | 144 | Control | 7 | Y | 0 | 0 |
| Wood Frog | 6 | Carter Meadow | 7 | Y | 0 | 153.60 |
| Wood Frog | 31 | Carter Meadow | 3 | Y | 0 | 103.47 |
| Wood Frog | 56 | Carter Meadow | 7 | Y | 0 | 0 |
| Wood Frog | 57 | Carter Meadow | 7 | Y | 0 | 0 |
| Wood Frog | 69 | Carter Meadow | 10 | Y | 0 | 164.80 |
| Wood Frog | 76 | Carter Meadow | 10 | Y | 0 | 624.00 |
| Wood Frog | 109 | Carter Meadow | 10 | Y | 0 | 155.20 |
| Wood Frog | 141 | Carter Meadow | 7 | Y | 0 | 331.20 |
| Wood Frog | 140 | Carter Meadow | 3 | Y | 3 | 174.93 |
| Wood Frog | 20 | Carter Meadow | 10 | Y | 3 | 161.60 |
| Wood Frog | 65 | Carter Meadow | 3 | Y | 5 | 68.80 |
| Wood Frog | 63 | Carter Meadow | 3 | Y | 6 | 32.53 |
| Wood Frog | 12 | Carter Meadow | 10 | Y | 7 | 91.20 |
| Wood Frog | 8 | Section Line | 10 | Y | 9 | 271.47 |
| Wood Frog | 89 | Section Line | 7 | Y | 25 | 176.00 |
| Wood Frog | 101 | Section Line | 7 | Y | 27 | 822.40 |
| Wood Frog | 11 | Section Line | 10 | N | 32 | 5492.80 |
| Wood Frog | 147 | Section Line | 10 | Y | 32 | 3242.13 |
| Wood Frog | 35 | Section Line | 9 | N | 48 | 2929.60 |
| Wood Frog | 145 | Section Line | 17 | N | 57 | 7406.40 |
| Wood Frog | 48 | Section Line | 3 | Y | 67 | 0 |
| Wood Frog | 40 | Section Line | 7 | Y | 72 | 708.80 |
| Wood Frog | 87 | Section Line | 3 | Y | 80 | 104.00 |
| Wood Frog | 129 | Section Line | 10 | Y | 81 | 4554.67 |
| Wood Frog | 47 | Section Line | 3 | Y | 85 | 20.80 |
| Wood Frog | 148 | Section Line | 10 | Y | 90 | 10176.53 |
| Wood Frog | 102 | Section Line | 5 | N | 93 | 806.40 |
| Wood Frog | 33 | Section Line | 6 | N | 95 | 1129.60 |
| Wood Frog | 60 | Section Line | 6 | N | 106 | 2768.00 |
| Wood Frog | 131 | Section Line | 7 | Y | 123 | 3184.00 |
| Wood Frog | 111 | Section Line | 10 | N | 127 | 12924.80 |
| Wood Frog | 42 | Section Line | 6 | N | 128 | 1427.20 |
| Wood Frog | 22 | Section Line | 8 | N | 128 | 6190.40 |
| Wood Frog | 10 | Section Line | 7 | Y | 136 | 2916.80 |
| Wood Frog | 37 | Section Line | 6 | N | 139 | 2632.00 |
| Wood Frog | 24 | Section Line | 3 | Y | 140 | 427.20 |
| Wood Frog | 107 | Section Line | 6 | N | 143 | 812.80 |
| Wood Frog | 116 | Section Line | 7 | N | 151 | 2308.80 |
| Wood Frog | 139 | Section Line | 6 | N | 156 | 1710.40 |
| Wood Frog | 106 | Section Line | 5 | N | 158 | 316.80 |
| Wood Frog | 68 | Section Line | 6 | N | 160 | 3116.80 |
| Wood Frog | 49 | Section Line | 6 | N | 164 | 2606.40 |
| Wood Frog | 130 | Section Line | 6 | N | 197 | 5881.60 |
| Wood Frog | 38 | Section Line | 5 | N | 227 | 195.20 |
| Wood Frog | 64 | Section Line | 6 | N | 228 | 1539.20 |
| Wood Frog | 91 | Section Line  (Prev. Exposed) | 7 | Y | 0 | 120.00 |
| Wood Frog | 127 | Section Line  (Prev. Exposed) | 3 | Y | 0 | 33.60 |
| Wood Frog | 136 | Section Line  (Prev. Exposed) | 7 | Y | 14 | 307.20 |
| Wood Frog | 97 | Section Line  (Prev. Exposed) | 7 | Y | 15 | 348.80 |
| Wood Frog | 81 | Section Line  (Prev. Exposed) | 3 | Y | 39 | 345.60 |
| Wood Frog | 46 | Section Line  (Prev. Exposed) | 4 | N | 110 | 1614.40 |

**Table S4.** Gene ontology (GO) term enrichment analysis for differentially expressed host contigs across amphibian species and *Bd* exposure treatments. Differential expression analyses were conducted in ‘edgeR’, and GO term enrichment was performed using ‘GOstats’. We compared all *Bd* exposure treatment samples to time-matched control samples for differential expression calling. For GO term enrichment analyses, we then pooled contigs within species and treatment groups showing a given response to exposure (i.e., up- or downregulation) at any time point. The top enriched GO terms (up to 10) within these contig sets are shown.

| **Species** | **Treatment** | **Direction of Differential Expression** | **No. of Differentially Expressed Contigs**  **(No. with GO Annotation)** | **No. of Enriched GO Terms** | **Top Enriched GO Terms** |
| --- | --- | --- | --- | --- | --- |
| Wood Frog | Carter Meadow  (Day 3, 7 or 10) | Up | 367  (150) | 342 | [1] cell envelope organization  [2] peptide cross-linking  [3] protein tetramerization  [4] keratinization  [5] hepoxilin biosynthetic process  [6] antigen processing and presentation  [7] lipoxygenase pathway  [8] antigen processing and presentation of peptide antigen via MHC class I  [9] immune response  [10] establishment of skin barrier |
| Wood Frog | Carter Meadow  (Day 3, 7, or 10) | Down | 131  (53) | 86 | [1] vacuolar sequestering  [2] diaphragm contraction  [3] digestion  [4] neuromuscular process controlling posture  [5] regulation of the force of heart contraction  [6] skeletal muscle contraction  [7] muscle cell cellular homeostasis  [8] aflatoxin B1 metabolic process  [9] mycotoxin metabolic process  [10] organic heteropentacyclic compound metabolic process |
| Wood Frog | Section Line  (Day 3, 7, or 10) | Up | 2967  (1299) | 669 | [1] response to external biotic stimulus  [2] establishment of localization  [3] protein N-linked glycosylation via asparagine  [4] response to unfolded protein  [5] response to bacterium  [6] ATP hydrolysis coupled proton transport  [7] protein glycosylation  [8] antigen processing and presentation of peptide antigen via MHC class I  [9] SRP-dependent cotranslational protein targeting to membrane  [10] glycosylation |
| Wood  Frog | Section Line  (Day 3, 7, or 10) | Down | 2792  (1169) | 347 | [1] collagen fibril organization  [2] regulation of cardiac muscle cell action potential involved in regulation of contraction  [3] bundle of His cell-Purkinje myocyte adhesion involved in cell communication  [4] regulation of blood circulation  [5] regulation of ventricular cardiac muscle cell action potential  [6] regulation of actin filament-based movement  [7] regulation of heart rate by cardiac conduction  [8] regulation of cardiac muscle contraction  [9] cellular amide metabolic process  [10] peptide cross-linking |
| Wood  Frog | PE Section Line  (Day 3 or 7) | Up | 1232  (523) | 665 | [1] response to external biotic stimulus  [2] antigen processing and presentation of peptide antigen via MHC class I  [3] immune response  [4] antigen processing and presentation  [5] tumor necrosis factor-mediated signaling pathway  [6] response to tumor necrosis factor  [7] innate immune response-activating signal transduction  [8] response to bacterium  [9] regulation of leukocyte activation  [10] positive regulation of immune response |
| Wood  Frog | PE Section Line  (Day 3 or 7) | Down | 1016  (454) | 358 | [1] regulation of cardiac muscle cell action potential involved in regulation of contraction  [2] bundle of His cell-Purkinje myocyte adhesion involved in cell communication  [3] regulation of ventricular cardiac muscle cell action potential  [4] regulation of cardiac muscle contraction  [5] muscle organ morphogenesis  [6] regulation of actin filament-based movement  [7] protein localization to adherens junction  [8] regulation of blood circulation  [9] regulation of action potential  [10] desmosome organization |
| American Bullfrog | Carter Meadow  (Day 3) | Up | 6  (1) | 1 | [1] glutathione metabolic process |
| American Bullfrog | Carter Meadow  (Day 3) | Down | 0  (N/A) | N/A | N/A |
| American  Bullfrog | Section Line  (Day 3, 7, or 10) | Up | 49  (19) | 62 | [1] arginine biosynthetic process  [2] urea cycle  [3] nitrogen cycle metabolic process  [4] keratinization  [5] cornea development in camera-type eye  [6] cyclic threonylcarbamoyladenosine biosynthetic process  [7] UDP-glucose metabolic process  [8] glucose 1-phosphate metabolic process  [9] UDP-glucuronate biosynthetic process  [10] negative regulation of mitochondrial membrane permeability |
| American Bullfrog | Section Line  (Day 3, 7, or 10) | Down | 10  (1) | 17 | [1] vitamin K metabolic process [2] cyclooxygenase pathway  [3] prostanoid biosynthetic process [4] prostaglandin metabolic process  [5] fatty acid derivative biosynthetic process  [6] drug metabolic process  [7] vitamin metabolic process [8] long-chain fatty acid metabolic process  [9] icosanoid metabolic process [10] fatty acid biosynthetic process |

**Table S5.** Differential expression results for *Bd* contigs recovered from wood frog samples. Differential expression analyses were conducted in ‘edgeR’ using a pathogen-specific RNA-seq dataset (i.e., composed only of *Bd* contigs). Treatment comparisons were specified for samples within species, and only results from wood frogs are shown as there was no differential expression detected in *Bd* contigs recovered from American bullfrog samples. Contig IDs are derived from the *Bd* reference transcriptome, and contig descriptions come from the Trinotate annotation pipeline.

| **Treatment Comparison** | **Direction of Differential Expression** | **No. of Differentially Expressed Contigs** | **Contig ID** | **Log2**  **Fold Change** | **Description** |
| --- | --- | --- | --- | --- | --- |
| SL vs. CM  (Day 3) | Up | 16 | [1] BDET_01382  [2] BDET_00873  [3] BDET_05372  [4] BDET_05630  [5] BDET_01380  [6] BDET_03287  [7] BDET_05531  [8] BDET_04763  [9] BDET_01885  [10] BDET_05222  [11] BDET_03743 [12] BDET_03866  [13] BDET_02997  [14] BDET_01124  [15] BDET_04800  [16] BDET_01379 | [1] 3.84  [2] 5.34  [3] 3.70  [4] 3.59  [5] 3.20  [6] 3.62  [7] 4.36  [8] 3.89  [9] 2.84  [10] 2.98 [11] 3.27  [12] 2.58 [13] 2.81  [14] 2.31 [15] 2.43 [16] 2.37 | [1] N/A  [2] ADP, ATP carrier protein 1, mitochondrial  [3] Uncharacterized threonine-rich GPI-anchored glycoprotein PJ4664.02  [4] ATP synthase subunit beta, mitochondrial  [5] N/A  [6] Adenosylhomocysteinase  [7] N/A  [8] Uncharacterized amino acid permease YfnA  [9] 5-methyltetrahydropteroyltriglutamate--homocysteine methyltransferase  [10] Heat shock protein 83  [11] N/A  [12] S-antigen protein  [13] ATP synthase subunit alpha, mitochondrial  [14] Heat shock protein HSS1  [15] N/A  [16] N/A |
| SL vs. CM  (Day 3) | Down | 1 | [1] BDET_06202 | [1] -2.75 | [1] Uncharacterized GTP-binding protein YGR210C |
| SL vs. CM  (Day 7) | Up | 0 | N/A | N/A | N/A |
| SL vs. CM  (Day 7) | Down | 0 | N/A | N/A | N/A |
| SL vs. CM  (Day 10) | Up | 0 | N/A | N/A | N/A |
| SL vs. CM  (Day 10) | Down | 0 | N/A | N/A | N/A |
| PE SL vs. SL  (Day 3) | Up | 0 | N/A | N/A | N/A |
| PE SL vs. SL  (Day 3) | Down | 14 | [1] BDET_01885  [2] BDET_05630  [3] BDET_04763  [4] BDET_01379  [5] BDET_05372  [6] BDET_01124  [7] BDET_03287  [8] BDET_04591  [9] BDET_01382 [10] BDET_03743  [11] BDET_01380 [12] BDET_02997  [13] BDET_03866 [14] BDET_05531 | [1] -3.80  [2] -3.19  [3] -3.88  [4] -3.12 [5] -2.87  [6] -2.86 [7] -3.24 [8] -4.22 [9] -2.40 [10] -3.21  [11] -2.50  [12] -2.76  [13] -2.52 [14] -2.97 | [1] 5-methyltetrahydropteroyltriglutamate--homocysteine methyltransferase  [2] ATP synthase subunit beta, mitochondrial  [3] Uncharacterized amino acid permease YfnA  [4] N/A  [5] Uncharacterized threonine-rich GPI-anchored glycoprotein PJ4664.02  [6] Heat shock protein HSS1  [7] Adenosylhomocysteinase  [8] N/A  [9] N/A  [10] N/A  [11] N/A  [12] ATP synthase subunit alpha, mitochondrial  [13] S-antigen protein  [14] N/A |
| PE SL vs. SL  (Day 7) | Up | 1 | [1] BDET_04147 | [1] 4.85 | [1] 40S ribosomal protein S2 |
| PE SL vs. SL  (Day 7) | Down | 1 | [1] BDET_01382 | [1] -6.55 | [1] N/A |

**Table S6.** Gene ontology (GO) term enrichment analysis for *Bd* contigs differentially expressed within wood frog samples. Differential expression analyses were conducted in ‘edgeR’ using a pathogen-specific RNA-seq dataset (i.e., composed only of *Bd* contigs). Treatment comparisons were specified for samples within species, and only results from wood frogs are shown as there was no differential expression detected in *Bd* contigs recovered from American bullfrog samples. GO term enrichment was performed using ‘GOstats’, and the top enriched GO terms (up to 10) within each contig set are shown.

| **Treatment Comparison** | **Direction of Differential Expression** | **No. of Differentially Expressed Contigs**  **(No. with GO Annotation)** | **No. of Enriched GO Terms** | **Top Enriched GO Terms** |
| --- | --- | --- | --- | --- |
| SL vs. CM  (Day 3) | Up | 16  (10) | 33 | [1] transmembrane transport  [2] ribonucleoside metabolic process  [3] purine nucleoside metabolic process  [4] single-organism localization  [5] glycosyl compound metabolic process  [6] ion transport  [7] inorganic ion transmembrane transport  [8] cation transmembrane transport  [9] ribonucleoside triphosphate metabolic process  [10] nucleoside biosynthetic process |
| SL vs. CM  (Day 3) | Down | 1  (1) | 0 | N/A |
| SL vs. CM  (Day 7) | Up | 0  (N/A) | N/A | N/A |
| SL vs. CM  (Day 7) | Down | 0  (N/A) | N/A | N/A |
| SL vs. CM  (Day 10) | Up | 0  (N/A) | N/A | N/A |
| SL vs. CM  (Day 10) | Down | 0  (N/A) | N/A | N/A |
| PE SL vs. SL  (Day 3) | Up | 0  (N/A) | N/A | N/A |
| PE SL vs. SL  (Day 3) | Down | 14  (8) | 32 | [1] ribonucleoside metabolic process  [2] purine nucleoside metabolic process  [3] glycosyl compound metabolic process  [4] ion transport  [5] transmembrane transport  [6] inorganic ion transmembrane transport  [7] cation transmembrane transport  [8] ribonucleoside triphosphate metabolic process  [9] nucleoside biosynthetic process  [10] purine ribonucleoside monophosphate biosynthetic process |
| PE SL vs. SL  (Day 7) | Up | 1  (1) | 0 | N/A |
| PE SL vs. SL  (Day 7) | Down | 1  (0) | N/A | N/A |
